# Inflammatory signature of cerebellar neurodegeneration during neonatal hyperbilirubinemia in *Ugt1*^*-/-*^ mouse model

**DOI:** 10.1186/s12974-017-0838-1

**Published:** 2017-03-24

**Authors:** Simone Vodret, Giulia Bortolussi, Jana Jašprová, Libor Vitek, Andrés F. Muro

**Affiliations:** 1 International Centre for Genetic Engineering and Biotechnology (ICGEB), Padriciano, 99, 34149 Trieste, Italy; 2 Institute of Medical Biochemistry and Laboratory Medicine, First Faculty of Medicine, Charles University, 120 00 Prague, Czech Republic; 3 Fourth Department of Internal Medicine, First Faculty of Medicine, Charles University, 120 00 Prague, Czech Republic

**Keywords:** Ugt1a1, Crigler-Najjar syndrome, Astrocytes, Microglia, Oxidative stress, Autophagy, Apoptosis, ER stress

## Abstract

**Background:**

Severe hyperbilirubinemia is toxic during central nervous system development. Prolonged and uncontrolled high levels of unconjugated bilirubin lead to bilirubin-induced neurological damage and eventually death by kernicterus. Bilirubin neurotoxicity is characterized by a wide array of neurological deficits, including irreversible abnormalities in motor, sensitive and cognitive functions, due to bilirubin accumulation in the brain. Despite the abundant literature documenting the in vitro and in vivo toxic effects of bilirubin, it is unclear which molecular and cellular events actually characterize bilirubin-induced neurodegeneration in vivo*.*

**Methods:**

We used a mouse model of neonatal hyperbilirubinemia to temporally and spatially define the response of the developing cerebellum to the bilirubin insult.

**Results:**

We showed that the exposure of developing cerebellum to sustained bilirubin levels induces the activation of oxidative stress, ER stress and inflammatory markers at the early stages of the disease onset. In particular, we identified TNFα and NFKβ as key mediators of bilirubin-induced inflammatory response. Moreover, we reported that M1 type microglia is increasingly activated during disease progression.

Failure to counteract this overwhelming stress condition resulted in the induction of the apoptotic pathway and the generation of the glial scar. Finally, bilirubin induced the autophagy pathway in the stages preceding death of the animals.

**Conclusions:**

This study demonstrates that inflammation is a key contributor to bilirubin damage that cooperates with ER stress in the onset of neurotoxicity. Pharmacological modulation of the inflammatory pathway may be a potential intervention target to ameliorate neonatal lethality in *Ugt1* ^*-/-*^ mice.

**Electronic supplementary material:**

The online version of this article (doi:10.1186/s12974-017-0838-1) contains supplementary material, which is available to authorized users.

## Background

Neonatal unconjugated hyperbilirubinemia is a common condition occurring in more than 60% of term newborns [1, 2] and almost all pre-term babies [3]. It is the result of the increased bilirubin production, mainly due to the high turnover of erythrocytes occurring after birth, and the delayed induction of the Ugt1a1 enzyme. It is usually considered a benign condition, but other concomitant causes may lead to uncontrolled acute hyperbilirubinemia [4, 5]. Prolonged high bilirubin levels are life threatening and often lead to permanent brain damage and death by kernicterus [6].

The incidence of kernicterus increases in underdeveloped and developing countries [5], and death by kernicterus is ranked as one of the three top causes of death among African newborns [7]. Clearly, the detailed identification of the neurological events and molecular targets triggering bilirubin neurotoxicity will help the understanding and management of this disease. To this aim, many in vitro and in vivo studies were carried out to increase the understanding of bilirubin neurotoxicity (for a review see [8]).

It has been shown that the free fraction of unbound bilirubin (Bf) causes neurotoxicity in vivo, and that the decrease of Bf by albumin supplementation increases neuro-protection and survival of the mutant mice and rats [9, 10].

Several studies using primary culture of neurons, glia cells or astrocytes, and neuronal-derived cell lines, showed that the sensitivity to bilirubin differs among cell types [11–14]. Moreover, bilirubin toxicity manifests in particular brain developmental stages, with less differentiated/immature neurons being more susceptible [15]. We showed in the *Ugt1* ^*-/-*^ mice that neurotoxicity is cell specific, being the Purkinje cells (PCs) the most affected neuron in the cerebellum [16–18].

By differential proteomic analysis of affected cerebella, we showed that bilirubin affects antioxidant defences reducing the overall capacity of neurons to tackle toxicity [18]. This observation was in line with in vitro experiments using primary cultures of neurons [19] and immortalized hepatoma cell lines [20, 21].

In addition, toxic levels of bilirubin induce an inflammatory response through the release of pro-inflammatory cytokines and the activation of MAPK pathway in astrocytes and microglial cells primary cultures [22, 23], leading to cell death. Moreover, high bilirubin levels induce oxidative stress and activate the P38 MAPK pathway in vivo and in vitro [18, 24, 25]. Likewise, in a Ugt1a humanized mouse model, Yueh et al showed that glia activation and oxidative stress are hallmarks of bilirubin-induced toxicity, being Toll-like receptor 2 (TLR2) a key player of bilirubin-mediated inflammatory response [26].

However, a time course study of the molecular events leading to bilirubin neurotoxicity in vivo is still missing. To this aim, we took advantage of the mouse model of severe neonatal hyperbilirubinemia generated in our lab [16, 17]. *Ugt1* ^*-/-*^ mice reproduce the major features of neonatal hyperbilirubinemia. In fact, consequent to the absence of Ugt1a1 bilirubin-glucuronidation activity in *Ugt1* ^*-/-*^ mice [16], total plasma bilirubin levels rise immediately after birth leading to important cerebellar damage, cerebellar hypoplasia and early death by kernicterus, with 50% mortality at post-natal day 11 (P11) and no survivors after P14 [17].

Using temporary phototherapy (PT) treatment during the neonatal period, we identified a critical window of neuronal susceptibility to bilirubin toxicity, corresponding to post-natal day 8 [17]. We showed that the degree of bilirubin damage correlates with the developmental stage of the cerebellum, being the early phases the more susceptible.

Based on the previous findings, in this study, we made a step forward aiming to investigate the temporal progression of the molecular and cellular events leading to bilirubin-induced neurodegeneration in the *Ugt1* ^*-/-*^ mouse model of neonatal hyperbilirubinemia.

## Methods

### Animals

Mice were housed and handled according to institutional guidelines, and experimental procedures approved by the ICGEB board, with full respect to the EU Directive 2010/63/EU for animal experimentation. *Ugt1* ^*-/-*^ mice in the FVB/NJ background have been generated previously [17]. Homozygous mutant animals were obtained from heterozygous mating. Male and female pups were used for these studies. WT littermates were used as control. Animals used in this study were at least 99.8% FVB/NJ genetic background, obtained after more than ten backcrosses with wild-type FVB/NJ mice. Mice were kept in a temperature-controlled environment with 12/12-h light/dark cycle. They received a standard chow diet and water ad libitum.

### Plasma bilirubin and UCB content measurement

Blood samples were collected at different time points in mutant and WT littermates by decapitation in EDTA-collecting tubes, at the moment of sacrificing the animals, as previously described [17]. Total bilirubin (TB) determination in plasma was performed using Direct and Total Bilirubin Reagent kit (BQ Kits, San Diego, CA) adapting the method to use minimal volumes (10 μl of plasma), as previously described [17].

Tissues for bilirubin content determination were collected and analysed as previously described [27].

### Preparation of total RNA from the mouse cerebellum and real-time PCR analysis

Total RNA from mouse cerebellum was prepared using EuroGOLD Trifast (Euroclone, Milano, Italy). One microgram of total RNA was reverse-transcribed, as previously described [18]. Total cDNA (1 μL) was used to perform qPCR using the specific primers listed in Additional file 1. qPCR was performed using the iQ SYBR Green Supermix (Bio-Rad) and a C1000 Thermal Cycler CFX96 Real Time System (Bio-Rad). Expression of the gene of interest was normalized to the Gapdh housekeeping gene. Data were analysed using the ΔΔCt method. All primers are listed in Additional file 1.

### Preparation of total protein extracts and western blot analysis

Cerebella were dissected, homogenized in lysis solution buffer (150 mM NaCl, 1% NP-40, 0.5% DOC, 0.1% SDS, 50 mM Tris HCl pH 8, 2× protease inhibitors) and analysed by western blot as described previously [16]. Primary antibodies used were as follows: anti-calbindin 1:2000 (Synaptic Systems, Goettingen, Germany), anti-NeuN 1:2000 (Millipore, Temecula, CA), anti-Glial Fibrillary acidic protein (GFAP) 1:1000 (Sigma, St. Louis, MO), anti-Iba1 1:1000 (Wako, Neuss, Germany), anti-cleaved caspase3 1:500 (Cell Signalling), anti-caspase3 1:800 (Santa Cruz Biotechnology), anti-CHOP 1:1000 (Santa Cruz, sc-56107), anti-HO1 1:1000 (Enzo Life Sciences, ADI-OSA-110) and anti-LC3 1:1000 (Sigma, L7543). Antibody anti-tubulin mAb E7 (Developmental Studies Hybridoma Bank, Iowa City, IA) or anti-Actin (Sigma, A2066) was used for loading control determination. Representative images are shown in the figures. Each timepoint was run in a separate gel.

### Brain histology and immunofluorescence

Nissl staining and immunofluorescence analysis of cerebellum samples were performed as previously described [17, 18]. The study was performed in a double-blind fashion: the genotype of the animals was unknown to the operator, while a different investigator analysed the data. Measurements were averaged for each animal.

Briefly, after blocking, specimens were incubated with the primary antibody for 2 h at RT in blocking solution with anti-calbindin 1:400 (Synaptic Systems, Goettingen, Germany), anti-NeuN 1:400 (Millipore, Temecula, CA), anti-glial fibrillary acidic protein, GFAP 1:200 (Sigma, St. Louis, MO), anti-iba1 1:200 (Wako, Neuss, Germany), anti-TNFα 1:100 (sc-1350, Santa Cruz), anti-NFKβ 1:100 (sc-8008, Santa Cruz), anti-MRC1 1:100 (CD206 clone MR5D3, MCA2235GA, BioRad), anti-CD68 1:100 (clone FA-11, BioRad), anti-CHOP 1:100 (GADD153, sc-56107, Santa Cruz), anti-HO1 1:200 (Enzo Life Sciences, ADI-OSA-110), anti-Fas 1:100 (CD95, NB120-13550, Novus Biologicals) and anti-HO1 1:200 (ADI-OSA-110, Enzo Life Sciences). Antigen retrieval with sodium citrate pH6 was performed for CHOP and HO1 immuno-stainings prior to blocking solution step. After 3× 5-min washes with blocking solution, specimens were incubated with secondary antibody (Alexa Fluor 488 or 568; Invitrogen Carlsbad, CA) for 2 h at RT. Nuclei were visualized by addition of Hoechst (10 μg/ml, Invitrogen) for 5 min after secondary antibody solution. Nissl-stained sections were mounted in Eukitt (Fluka, St. Louis, MO, USA) while immunofluorescence (IF) slides were mounted in Mowiol 4-88 (Sigma-Aldrich). Images were acquired on a Nikon Eclipse E-800 epi-fluorescent microscope with a charge-coupled device camera (DMX 1200 F; Nikon Amstelveen, The Netherlands). Digital images were collected using ACT-1 (Nikon) software.

Analysis of the layer thickness was performed on Nissl-stained sections by measuring the layer depth (μm) as previously described [16]. PC density analyses were performed as previously described [16, 17].

### Statistics

The Prism package (GraphPad Software, La Jolla, CA) was used to analyse the data. Results are expressed as mean ± S.D. Values of *p <* 0.05 were considered statistically significant. Depending on the experiment, Student’s *t* test or two-way ANOVA, with Bonferroni’s post hoc comparison tests, were used, as indicated in the legends to the figures and text. Correlation analyses were done using the Pearson coefficient to assess the linearity between two variables and calculate two-tailed *p* value (95% of confidence interval).

## Results

To better dissect the mechanisms leading to bilirubin neurotoxicity, we analysed three sequential time points: P5 at the onset of the pathology; P8 as a more advanced pathological condition, in which lethality is partially reversible by phototherapy (PT) application in a low proportion of mice [17]; and finally P10, as the latest and most severe phase, just one day before reaching 50% mortality [17].

**Fig. 1:**
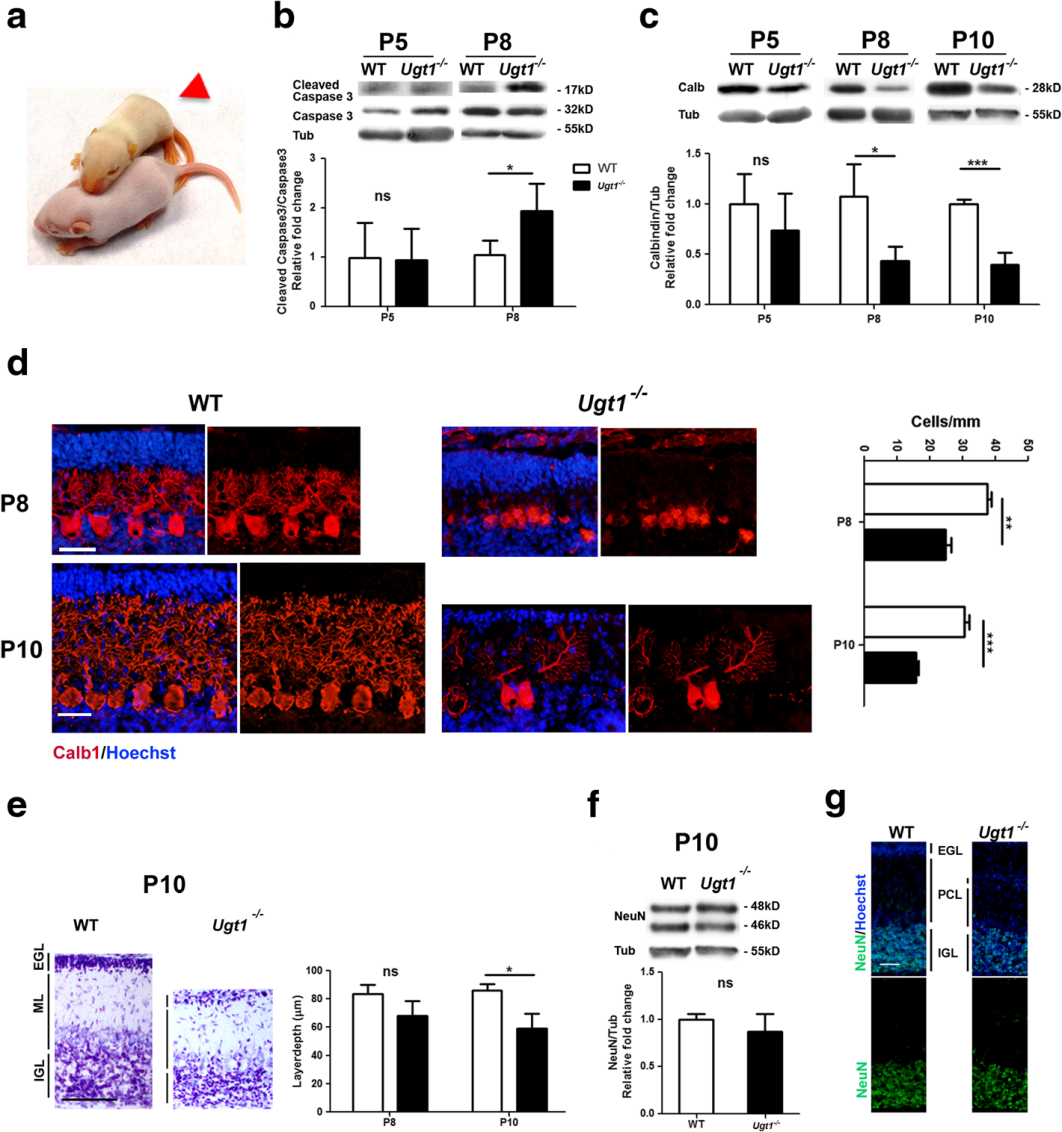
Cerebellar neurons are differentially vulnerable to bilirubin. **a** Appearance of a jaundiced *Ugt1* ^*-/-*^ mouse (*Ugt1* ^*-/-*^, *red arrowed*) and a wild-type (WT) littermate at P8. **b** WB analysis of total cerebellum protein extract using anti-cleaved and anti-total caspase3 antibodies at P5 and P8 of WT and *Ugt1* ^*-/-*^ mice. The bar graphs show the mean of cleaved/total caspase3 ratio of the bands. **c** WB analysis and quantification of total cerebellum protein extracts of WT and *Ugt1* ^*-/-*^ mice using an anti-calbindin antibody at the indicated time points. **d** Representative fluorescent immunohistochemistry of cerebellar sections from WT and *Ugt1* ^*-/-*^ mice at P8 and P10, using an anti-calbindin antibody (*red*) to highlight PCs. Hoechst (*blue*) was used to mark nuclei. *Scale bar* 50 μm. The quantification of PC number at P8 and P10 of WT and *Ugt1* ^*-/-*^ mice is represented in the bar graph (cell/mm). **e** *Left panel*, Nissl staining of cerebellar layers at P10 of WT and *Ugt1* ^*-/-*^ mice. *Scale bar* 100 μm. *Right panel*, layer depth quantification of P8 and P10 WT and *Ugt1* ^*-/-*^ (μm). **f** WB analysis of total cerebellum protein extracts of WT and *Ugt1* ^*-/-*^ mice at P10 using an anti-NeuN antibody. **g** Representative fluorescent immunohistochemistry of cerebellar sections from WT and *Ugt1* ^*-/-*^ mice using anti-NeuN antibody (*green*) to stain differentiated granule cells. Hoechst dye was used to stain nuclei (*blue*). *Scale bar* 50 μm. For all the experiments, values represent mean ± SD. Student’s *t* test, *p < 0.05, **p < 0.01, ***p < 0.001. The number of WT and *Ugt1* ^*-/-*^ was ≥3 in all the experiments and time points. For WB analysis, β-tubulin was used as loading control. *EGL* external germinal layer, *IGL* internal granular layer, *ML* molecular layer

### Vulnerability to bilirubin depends on the developmental stage and the neuronal cell type

We have previously reported that bilirubin induces apoptosis in the cerebellum of C57Bl/6 *Ugt1* ^*-/-*^ mice at P4 [18], a strain carrying the same genetic mutation but showing a more severe phenotype [16]. Here, we used a less severe mouse strain (Ugt1^-/-^/FVB/NJ) to study the apoptotic pathway in the cerebellum, which is the most vulnerable region to bilirubin toxicity in rodent animal models (Fig. 1a) [17, 28, 29]. No increase in caspase 3 cleavage was observed at P5 (Fig. 1b), but apoptosis occurred at P8, as demonstrated by a twofold increase of activated caspase 3 (Fig. 1b). These results are in line with our previous data showing that, despite some of the mice were rescued from mortality by PT starting at P8, the accumulated damage was not reversible [17].

Neurodegeneration is a well-known feature of bilirubin-induced neurological dysfunction (BIND). To have a deeper insight in the mechanisms leading to cerebellar hypoplasia, we first estimated by western blot (WB) analysis the levels of calbindin, a Purkinje cell (PC)-specific marker [30]. We observed that PCs were not affected at P5, while at P8 and P10, there was a significant reduction in calbindin levels in *Ugt1* ^*-/-*^ cerebellar protein extracts (56 and 61% decrease, at P8 and P10, respectively; Fig. 1c). Similarly, IF analysis showed a reduction in PC number of 30 and 50% at P8 and P10, respectively, and the almost complete disappearance of their dendritic arborization (Fig. 1d).

Likewise, we assessed the degree of cerebellar damage in brain sections of *Ugt1* ^*-/-*^ mice over the phases preceding death (Fig. 1e and Additional file 2A). The long-term exposure of cerebellar neurons to high levels of bilirubin resulted in an overall reduction in cerebellar cortex thickness of 20 and 32%, at P8 and P10, respectively (Fig. 1e). Layer-thickness quantification showed that the external germinal layer (EGL)- and molecular layer (ML)-thickness were significantly reduced at both P8 and P10, while the internal granular layer (IGL)-thickness was reduced only at P10 (Additional file 2A).

Next, we verified whether bilirubin affected mature cerebellar granule cells (GCs) by determining NeuN expression, a nuclear protein antigen whose expression in the cerebellum is restricted to differentiated granule neurons [31]. Western blot experiments showed that bilirubin had no evident effects on differentiated GCs in all time points analysed (Fig. 1f and Additional file 2B). IF analysis performed at P8 and P10 showed no obvious reduction in cell density of neurons located in the IGL (Fig. 1g and Additional file 2C, respectively), suggesting that differentiated GCs were not affected by chronic exposure to bilirubin.

Collectively, our data support the concept that vulnerability of different neuronal cell types in vivo is associated with their developmental stage.

**Fig. 2:**
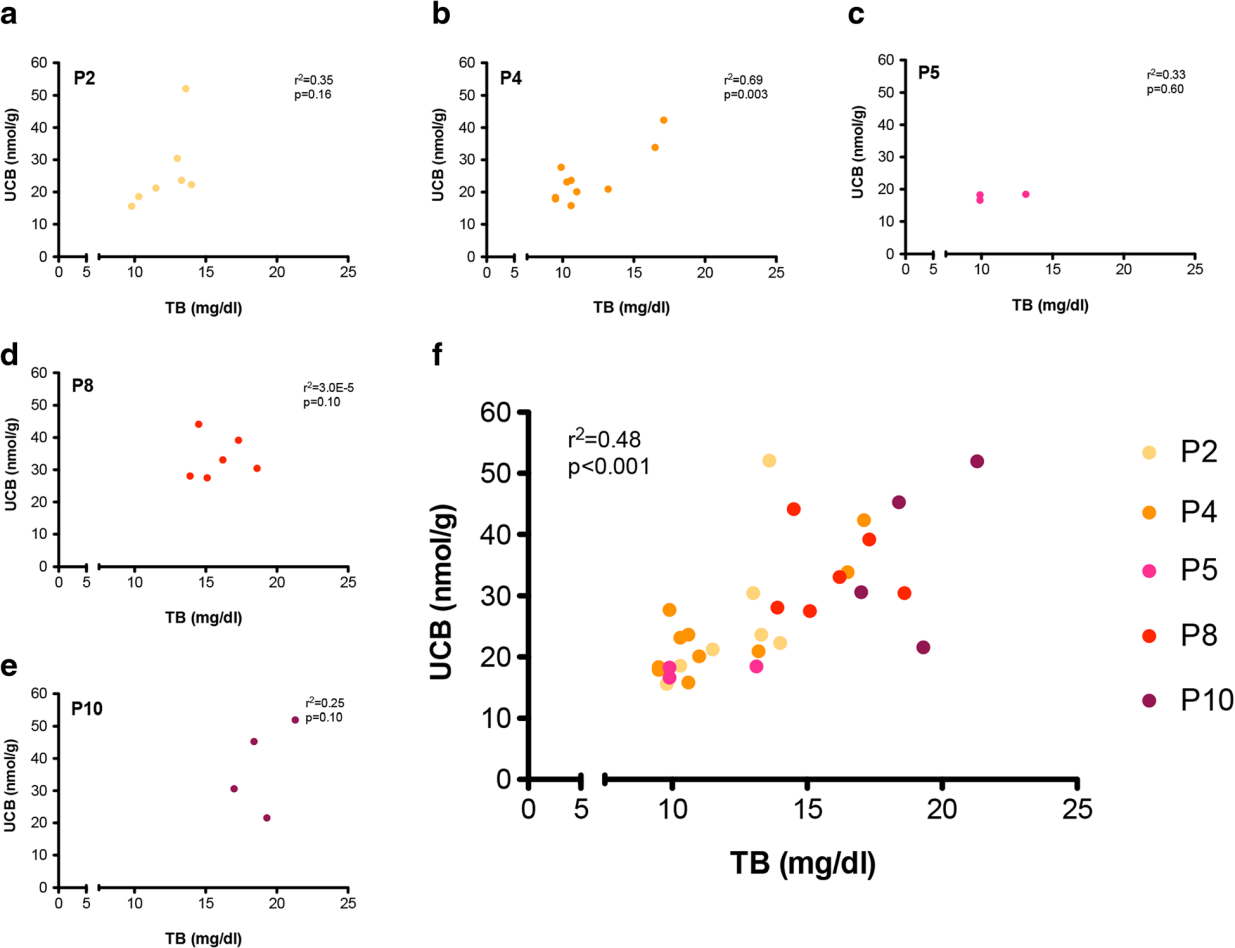
Correlation analysis between total plasma bilirubin and cerebellar bilirubin content at different post-natal ages. Plasma total bilirubin (mg/dL) and cerebellar UCB content (nmol/g) were plotted at each single time point: **a** P2, **b** P4, **c** P5, **d** P8 and **e** P10. **f** All time points in a single graph. *Each dot* represents a single animal. Correlation analyses were done using the Pearson coefficient to assess the linearity between two variables and calculate two-tailed *p* value (95% of confidence interval)

Then, we determined the levels of plasma and cerebellar bilirubin at different time points, ranging from P2 to P10 (Fig. 2). Mutant animals showed a time-dependent increase in plasma bilirubin levels, associated to the worsening of the phenotype. Importantly, there was a clear correlation between the levels of UCB in the cerebellum and those found in plasma (*p* < 0.001; Fig. 2f). These results demonstrate the tight association between the accumulated levels of UCB in the cerebellum and the increase in neurodegeneration and apoptosis.

### Bilirubin induces macro- and micro-gliosis in the cerebellum of *Ugt1*^*-/-*^ mice

Since neurotoxicity is normally associated with neuroinflammation [32, 33], we investigated whether bilirubin-induced cell death was accompanied by an increase of inflammatory markers in the cerebellum of the *Ugt1* ^*-/-*^ mice.

**Fig. 3:**
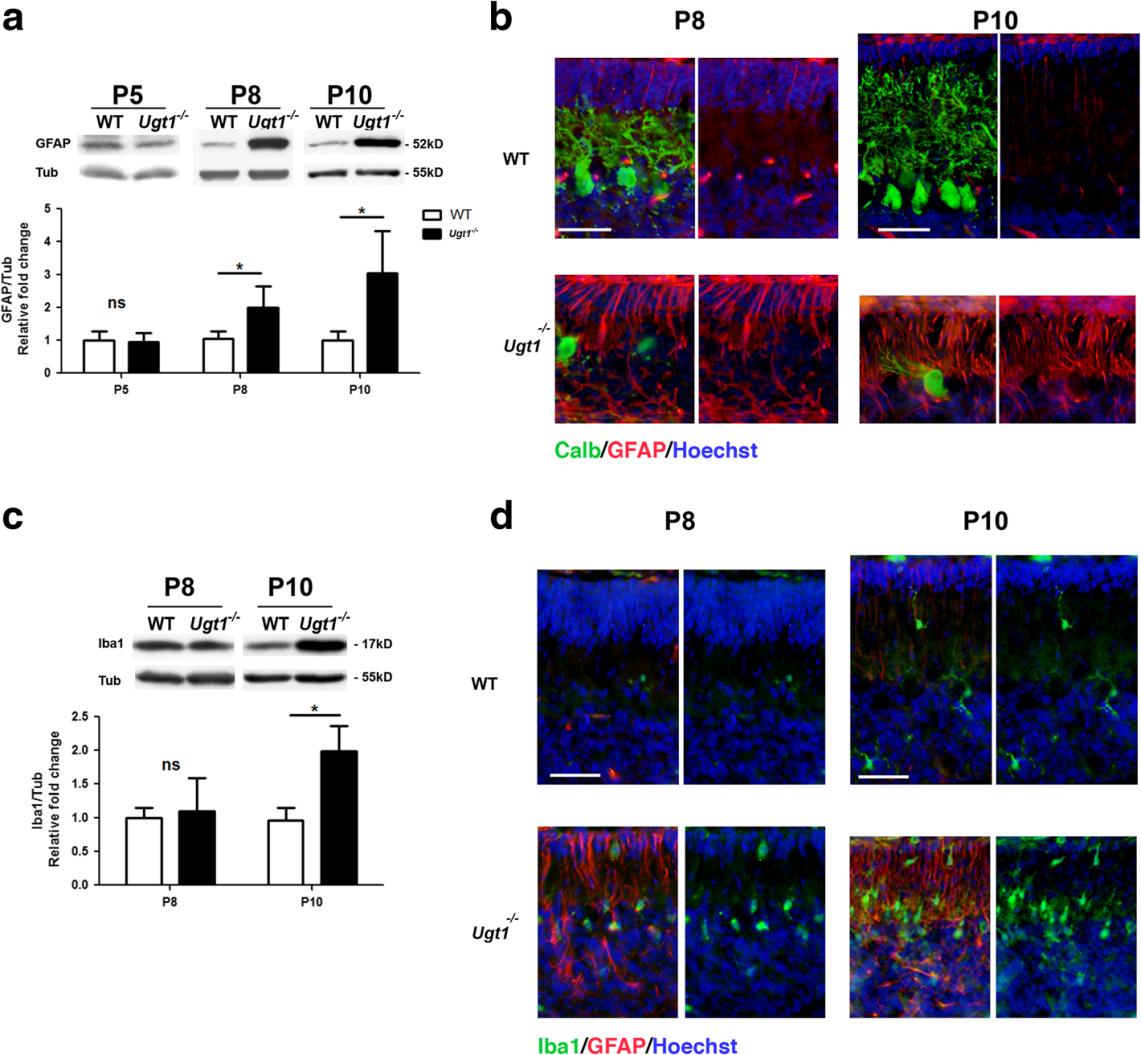
Bilirubin neurotoxicity activates astrocytes and microglia cells. **a** WB analysis of total cerebellum protein extract using an anti-GFAP antibody at indicated time points of WT and *Ugt1* ^*-/-*^ mice. **b** Representative fluorescent immunohistochemistry of cerebellar sections from WT and *Ugt1* ^*-/-*^ mice using anti-GFAP antibody (*red*) to detect astrocytes, co-stained with an anti-calbindin antibody (*green*) to highlight PCs. *Scale bar* 50 μm. **c** WB analysis of total cerebellum protein extracts using an anti-Iba1 antibody at P8 and P10 of WT and *Ugt1* ^*-/-*^ mice. **d** Representative fluorescent immunohistochemistry of cerebellar sections from WT and *Ugt1* ^*-/-*^ mice using an anti-Iba1 antibody (*green*) to detect microglia, co-stained with an anti-GFAP antibody (*red*) to highlight astrocytes. *Scale bar* 50 μm. For all the experiments, values represent the mean ± SD. Students *t* test, *p < 0.05. The number of WT and *Ugt1* ^*-/-*^ was ≥3 in all the experiments and time points. For WB analysis, β-tubulin was used as loading control. For IF, Hoechst dye (*blue*) was used to mark nuclei

First, we analysed astrocyte response to bilirubin, by determining glial fibrillary acidic protein (GFAP) levels, an astrocyte-specific marker. No astrocytosis was detected in the early time point of bilirubin exposure (P5, Fig. 3a). Conversely, GFAP levels were two and threefold increased at P8 and P10, respectively, as determined by WB analysis (P8 and P10, Fig. 3a).

IF analysis performed at P8 and P10 revealed that the glial scar was selectively localized in those cerebellar fissures where PC loss was more prominent (Fig. 3b), such as fissures IV to VIII (Additional file 3A, section of whole cerebellum at P10). To note, the time-dependent increase in GFAP protein levels was also evident at mRNA level (Additional file 3B). These finding were in good correlation with the time-dependent loss of PCs (Fig. 1d).

Next, we determined the cerebellar microglia response to bilirubin by the analysis of ionized calcium-binding adapter molecule 1 (Iba1) as cell-specific marker. We observed that bilirubin triggered an increase in *Iba1* mRNA levels starting from P8, and reaching up to threefold increase at P10, as determined by real-time RT-PCR (Additional file 4A). However, at protein levels, we observed a twofold increase in Iba1 only at P10 (Fig. 3c). Furthermore, IF staining of cerebellar sections showed that Iba1-positive cells were located in those zones more affected by bilirubin (Fig. 3d and Additional file 4B).

**Fig. 4:**
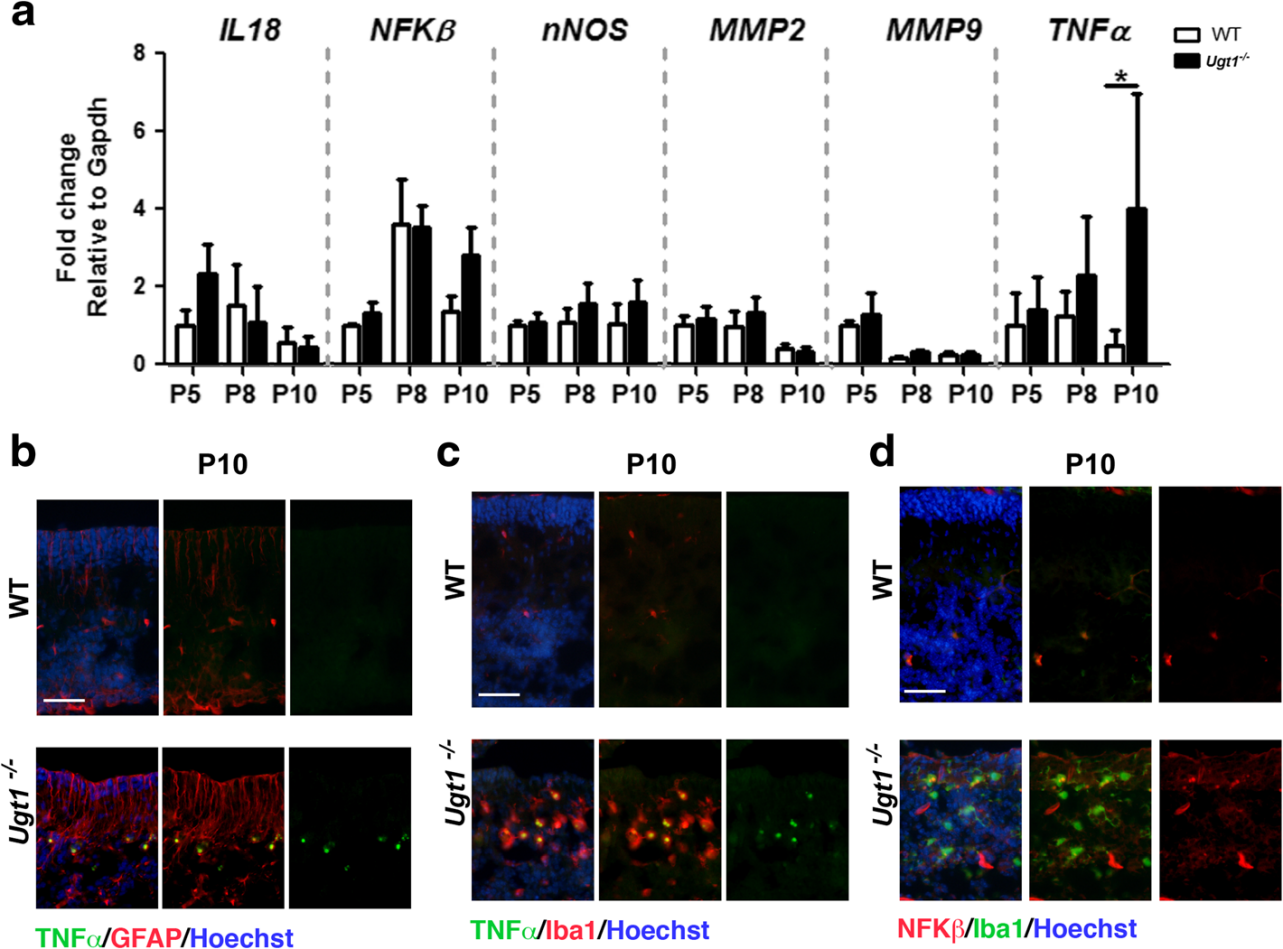
Inflammatory mediators of bilirubin neurotoxicity. **a** Relative mRNA expression of inflammatory markers. WT and *Ugt1* ^*-/-*^ mice mRNA cerebellar expression levels of *IL18*, *NFKβ*, *nNOS*, *MMP2*, *MMP9* and *TNFα* were analysed at the indicated time points by qRT-PCR. For each gene, data were normalized according to the values of the WT samples at P5. For all the experiments, values represent the mean ± SD. Two-way ANOVA test, *p < 0.05. The number of WT and *Ugt1* ^*-/-*^ was ≥3 in all the experiments and time points. **b** Representative IF of cerebellar sections from WT and *Ugt1* ^*-/-*^ mice at P10 using an anti-TNFα antibody (*green*), co-stained with an anti-GFAP antibody (*red*) to highlight astrocytes. **c** Representative IF of cerebellar sections at P10 using an anti-TNFα antibody (*green*), co-stained with an anti-Iba1 antibody (*red*) to highlight microglia. **d** Representative IF of cerebellar sections at P10 using an anti-NFKβ antibody (*red*), co-stained with an anti-Iba1 antibody (*green*) to highlight microglia. *Scale bar* 50 μm. For IF, Hoechst dye (*blue*) was used to mark nuclei

Interestingly, among all the inflammation markers tested (Fig. 4a and Additional file 5), only *TNFα* was differentially expressed in mutant animals compared to WT littermates, reaching up to ~10-fold (two-way ANOVA, *p* < 0.05) in very severe conditions, such as P10 (Fig. 4a). Immunofluorescence analysis of cerebellar sections at P10 showed the presence of TNFα- and NFKβ-positive cells in cerebellar samples from mutant mice, which co-stained with microglia-positive cells (Fig. 4b–d).

These data underline the complexity of the inflammatory response triggered by bilirubin in vivo and indicate that neuroinflammation is an important player contributing to each stage of the damage in our experimental model. Overall, these data show that the inflammatory response in vivo increases over time as bilirubin accumulates in the cerebellum and parallel the onset of neurodegeneration.

### Time-dependent decrease of M2 is inversely proportional to M1 activation

To better characterize the microglia activation during prolonged bilirubin exposure, we performed real-time RT-PCR analysis of the molecular markers that discriminate the activation of microglia/macrophage states, such as those characterizing the pro-inflammatory M1 microglia (*CD68* and *CD86*) and the anti-inflammatory M2 microglia (*MRC1*, *Arg1* and *MRC2*).

**Fig. 5:**
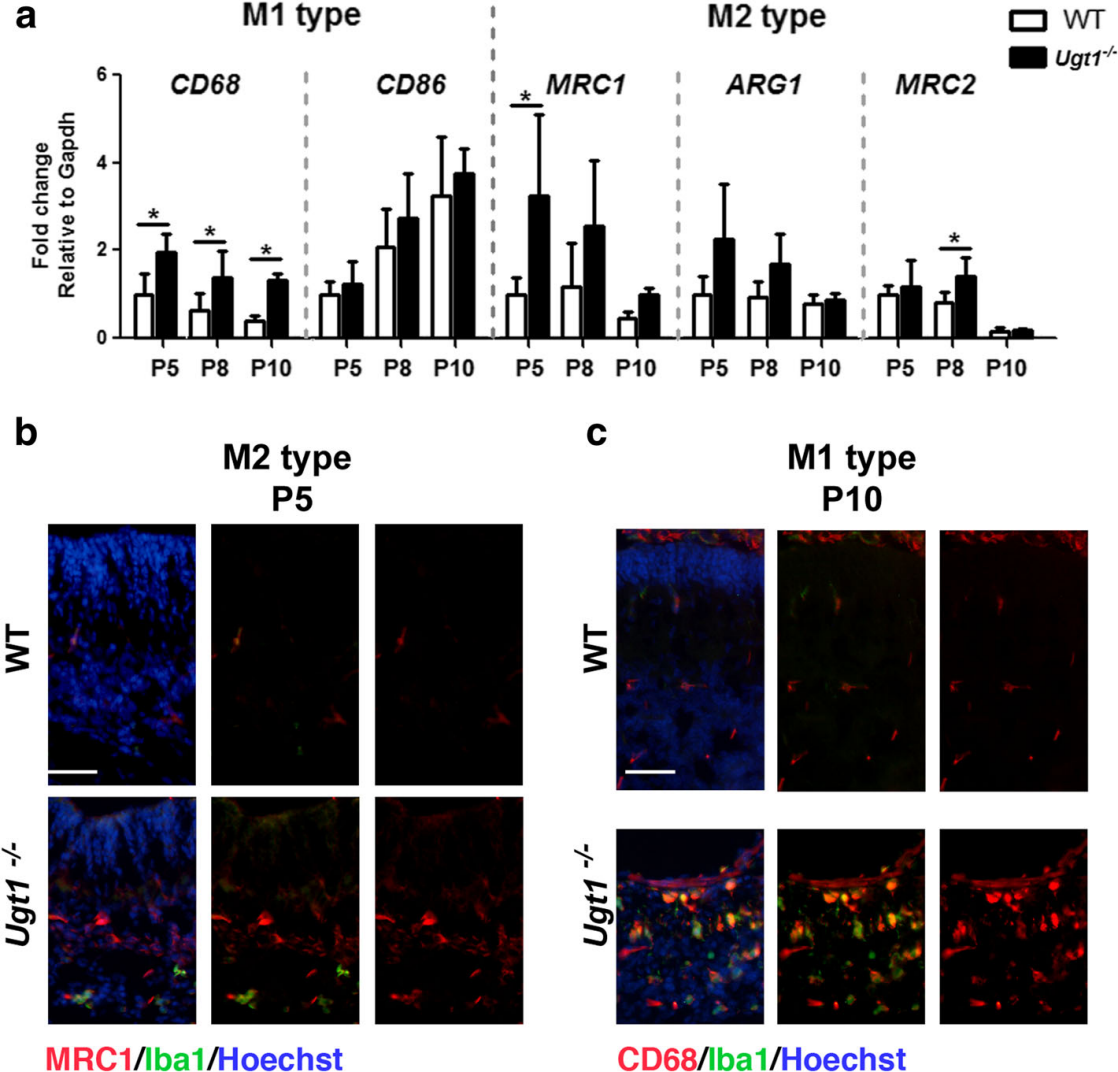
Bilirubin induces activation of M1 and M2 microglia. **a** Time course of mRNA expression levels of M1 and M2 microglia markers. WT and *Ugt1* ^*-/-*^ mice mRNA cerebellar expression levels of *CD68*, *CD86*, *MCR1*, *Arg1* and *MCR2* were analysed at the indicated time points by qRT-PCR. For each gene, data were normalized according to the values of the WT samples at P5. For all the experiments, values represent the mean ± SD. Two-way ANOVA test, *p < 0.05. The number of WT and *Ugt1* ^*-/-*^ was ≥3 in all the experiments and time points. **b** Representative IF of cerebellar sections from WT and *Ugt1* ^*-/-*^ mice at P5 using an anti-MRC1 antibody (*red*) to detect M2 microglia, co-stained with an anti-Iba1 antibody (*green*) to highlight microglia. **c** Representative IF of cerebellar sections at P10 using an anti-CD68 antibody (*red*) to detect M1 microglia, co-stained with an anti-Iba1 antibody (*green*). For IF, Hoechst dye (*blue*) was used to mark nuclei. *Scale bar* 50 μm

We observed that the messenger RNA (mRNA) of the M1 pro-inflammatory microglia marker *CD68* showed 2.0-, 2.5- and 4.0-fold increase at P5, P8 and P10, respectively, compared to WT samples at the same time points (Fig. 5a). Unexpectedly, when the mRNA expression of *CD86*, another component of M1 type microglia, was compared between *Ugt1* ^*-/-*^ mice and WT littermates, no upregulation was observed.

By contrast, real-time RT-PCR analysis revealed an inverted trend for the M2 type, which is considered to compose the neuroprotective counterpart of microglia. *MRC1* mRNA in *Ugt1* ^*-/-*^ mice was upregulated at P5 and showed decreased expression over time (the same pattern was observed for *Arg1*, but differences were not statistically significant), while *MRC2* was significantly activated only at P8, and mRNA levels decreased at P10, both in WT and Ugt1^-/-^ cerebella (Fig. 5a).

IF experiments reinforced the findings obtained by real-time PCR analysis showing MRC1-positive cells at P5 (Fig. 5b), and of CD68 at P10 (Fig. 5c). In particular, a portion of MRC1-positive cells co-stained with Iba1 at P5, confirming the activation of M2 microglia at the early stages of the disease. Likewise, we reported an important increase in CD68-positive cells at P10, providing evidence of pro-inflammatory M1 type of microglia activation at the later stages of the disease (Fig. 5c).

Since the transition from M2 to M1 phenotype is generally associated with inflammation-induced pathologies, taken together these data indicate that exposure to bilirubin triggers the activation of pro-inflammatory microglia with a parallel decrease in the neuroprotective one.

### ER stress and oxidative stress activation in the early stages

It has been shown that bilirubin is involved in endoplasmic reticulum (ER) stress response activation in in vitro cell cultures [34–36]. Thus, we investigated whether ER stress is a key event in the onset of bilirubin toxicity in vivo.

**Fig. 6:**
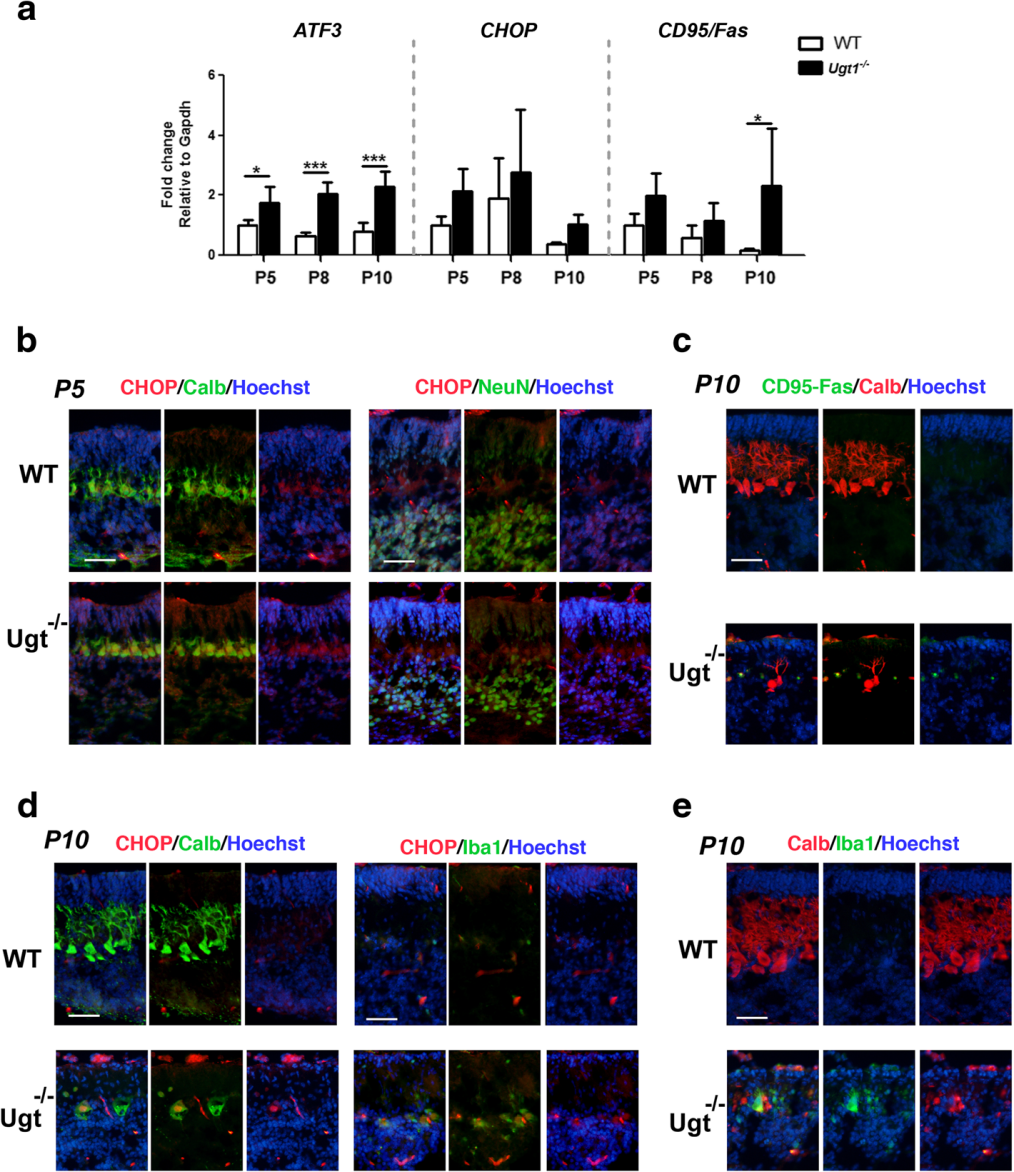
ER stress response to sustained bilirubin levels. **a** Relative mRNA expression analysis of different genes to assess the ER stress response over prolonged bilirubin exposure. WT and *Ugt1* ^*-/-*^ mice mRNA expression levels of *ATF3*, *CHOP* and *CD95/Fas* were analysed at indicated time points by qRT-PCR. For each gene, data were normalized according to the values of the WT samples at P5. For all the experiments values represent the mean ± SD. Two-way ANOVA test, *p < 0.05. The number of WT and *Ugt1* ^*-/-*^ was ≥3 in all the experiments and time points. **b** Representative IF of cerebellar sections from WT and *Ugt1* ^*-/-*^ mice at P5 using an anti-CHOP antibody (*red*), co-stained with (*left panel*) an anti-calbindin antibody (*green*) to highlight Purkinje cells or (*right panel*) anti-NeuN to highlight granule cells. **c** Representative IF of cerebellar sections at P10 using an anti-CD95/Fas antibody (*green*), an anti-calbindin antibody (*red*) to highlight Purkinje cells. **d** Representative IF of cerebellar sections at P10 using an anti-CHOP antibody (*red*), co-stained with (*left panel*) an anti-calbindin antibody (*green*) or (*right panel*) anti-Iba1 to highlight microglia. **e** Representative IF of cerebellar sections at P10 using an anti-Calbindin antibody (*red*), co-stained with an anti-Iba1 antibody (*green*). For IF, Hoechst dye (*blue*) was used to mark nuclei. *Scale bar* 50 μm

Concomitantly to the activation of inflammation, we observed that the bilirubin insult also triggered the activation of different ER stress markers during the early phase of hyperbilirubinemia, as determined by real-time RT-PCR analysis (Fig. 6a and Additional file 6A). mRNA levels of *ATF3, CD95/Fas* and CHOP increased as aggravating stages of bilirubin toxicity moved forward. Since CHOP is a key effector in ER stress response, we performed IF analysis of cerebellar sections at P5 and at P10. IF analysis showed that CHOP signal was increased in mutant mice compared to WT littermates in all cerebellar layers at P5 (external germinal layer, Purkinje cell layer and internal granular layer, Fig. 6b).

As neurodegeneration moved forward, we observed that Fas/CD95 and CHOP signal concentrated in the PCL (Fig. 6c, d). In particular, at P10, CHOP was mainly localized in the nucleus of severely neurodegenerated PCs, but not in granule cell neurons (Fig. 6d and Additional file 6D). Moreover, we observed that CHOP-positive Purkinje cells were surrounded by Iba1-positive cells (Fig. 6d, e and Additional file 6E) indicating that microglia cells were actively engulfing degenerated neurons.

**Fig. 7:**
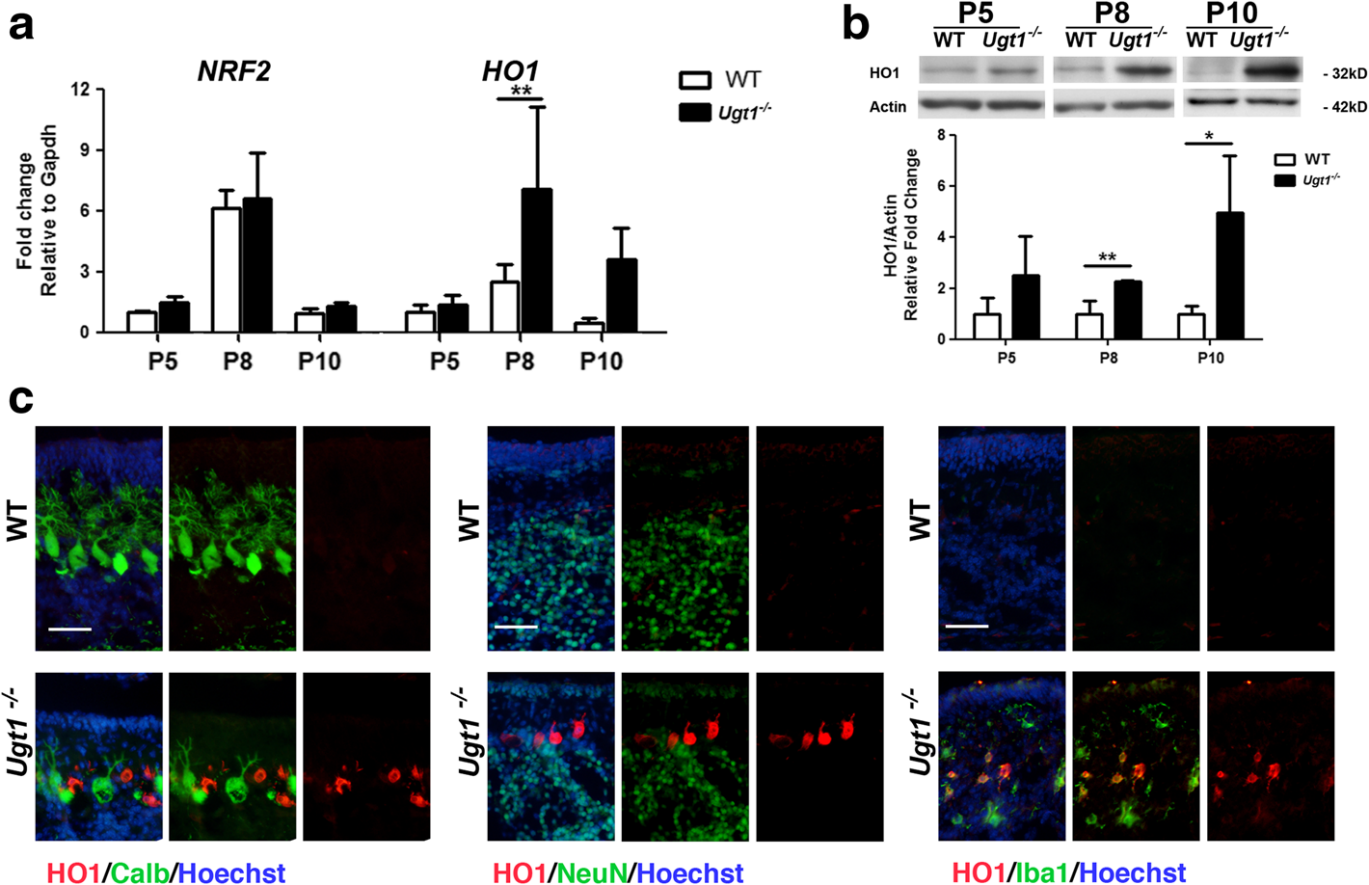
Bilirubin-induced oxidative stress response. **a** Relative mRNA expression of different genes to assess the oxidative stress response. Expression levels of *Nrf2* and *HO1* in WT and *Ugt1* ^*-/-*^ mice were analysed at P5, P8 and P10 by qRT-PCR. For each gene, data were normalized according to the values of the WT samples at P5. Values represent the mean ± S.D. Two-way ANOVA test, **p < 0.01. The number of WT and *Ugt1* ^*-/-*^ was ≥3 in all the experiments and time points. **b** WB analysis and quantification of total cerebellum protein extracts of WT and *Ugt1* ^*-/-*^ mice using an anti-HO1 antibody at the indicated time points. Actin was used as loading control. Student’s *t* test, *p < 0.05, **p < 0.01. **c** Representative IF of cerebellar sections from WT and *Ugt1* ^*-/-*^ mice at P10 using an anti-HO1 antibody (*red*), co-stained with (*left panel*) an anti-calbindin antibody (*green*) to highlight Purkinje cells, (*central panel*) anti-NeuN antibody to highlight granule cells or (*right panel*) anti-Iba1 antibody to highlight microglia. For IF, Hoechst dye (*blue*) was used to mark nuclei. *Scale bar* 50 μm

Finally, we focused our attention on oxidative stress, which is considered a major process triggered by bilirubin in vivo [18]. By real-time RT-PCR, different markers of oxidative stress were analysed to assess their activation status (Fig. 7a and Additional file 7A-B). We could not detect any variation in mRNA levels of the analysed cytochrome P450 family 1 subfamily A member 2 (*Cyp1a1*), and cytochrome P450 family 2 subfamily A member 5 (*Cyp2a5*) (Additional file 7B), nor in *Nrf2*, an important transcription factor involved in cellular response to oxidative stress (Fig. 7a). On the contrary, both the mRNA and protein expression levels of *HO1* (heme oxygenase 1), a key enzyme in heme catabolism, were increased in RNA and protein extracts from *Ugt1* ^*-/-*^ cerebella, in a time-dependent manner, starting from P8 and reaching more than fourfold increase at P10 at protein level (Fig. 7b). IF analysis showed an important increase of HO1-positive cells in the Purkinje cell area (P5 and P10, Additional file 7C and Fig. 7c, respectively). All HO1-positive cells were also Iba1 positive, but not Calbindin or NeuN positive, suggesting that some microglia cells experienced oxidative stress as a consequence of their phagocytic activity on degenerated PCs.

### Autophagy is a late event triggered by bilirubin

Because of the close connection between oxidative stress, neuro-apoptosis, inflammation and autophagy, we investigated whether the latter was activated in vivo in response to bilirubin.

**Fig. 8:**
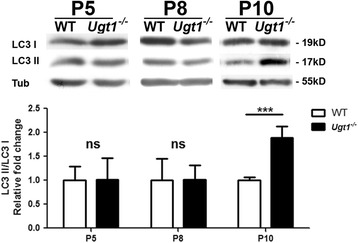
Bilirubin triggers autophagy in the cerebellum of *Ugt1* ^*-/-*^ mice. WB analysis of total cerebellum protein extracts using an anti-LC3 antibody at P5, P8 and P10 of WT and *Ugt1* ^*-/-*^ mice. β-tubulin was used as a loading control. *Lower panel*: densitometric quantification of the bands; results are expressed as the mean of the LC3 II/I ratio. Student’s *t* test, ***p < 0.001. *P5*: WT *n* = 4, *Ugt1* ^*-/-*^ *n* = 4; *P8*: WT *n* = 4, *Ugt1* ^*-/-*^ *n* = 4; *P10*: WT *n* = 3; *Ugt1* ^*-/-*^ *n* = 5

To determine whether autophagy is triggered by hyperbilirubinemia, we performed a WB analysis and quantified the levels of the LC3-II form, as a marker of autophagy. We observed the increase the LC3-II form only in cerebellar total extracts of *Ugt1* ^*-/-*^ animals at P10 (Fig. 8), corresponding to the most severe time point analysed.

These data showed, for the first time, that bilirubin triggers autophagy in vivo in a mouse model of severe neonatal hyperbilirubinemia.

## Discussion

Unveiling the mechanisms operating at the onset of a given disease is essential to develop potential pharmacological therapies against bilirubin-induced brain damage.

**Fig. 9:**
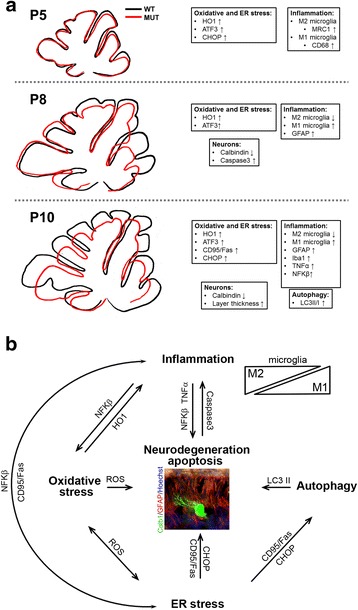
Molecular events leading to bilirubin neurodegeneration in a mouse model of neonatal hyperbilirubinemia. **a** The three sequential phases of the disease progression are indicated: *P5*, *P8* and *P10*. Representative cartoon showing the morphology of cerebellar sections from WT (black line) and *Ugt1* ^*-/-*^ (red line). Bilirubin affects cerebellar size of *Ugt1* ^*-/-*^ mice in a time-dependent manner. Boxes indicate the main pathways and molecular players regulated by bilirubin. **b** Proposed interplay between the molecular events leading to cerebellar neurodegeneration. Exposure of the developing cerebellum to toxic bilirubin levels triggers the activation of neuroinflammation, ER stress, oxidative stress and autophagy pathways. *Bold font* indicates main mechanisms involved in the onset of the disease; *arrows* and *molecular markers* indicate the interconnection between different mechanisms. An image of a neurodegenerated Purkinje neuron (stained with Calb1, *green*) surrounded by glial scar (stained with GFAP, *red*) is represented

Our data indicate that long-term exposure of the developing cerebellum to high bilirubin levels induces the concomitant activation of different mechanism, ranging from inflammation to ER stress. In particular, we observe that the inflammatory response plus ER stress are activated in the early stages of the disease onset and, in turn, they affect survival of developing neurons. At last, bilirubin-induced neurotoxicity stimulates the autophagy pathway (Fig. 9a).

In the following sections, we will discuss in detail the interplay between the major players identified in the present work (Fig. 9b) and provide possible future directions.

### Oxidative stress response

Although mildly elevated bilirubin concentrations are considered beneficial due to its antioxidant properties, high levels of bilirubin lead to considerable oxidative stress [37, 38]. This is particularly evident in the central nervous system when ROS production overwhelms antioxidant defences [39], resulting in neurodegeneration.

Typically, the redox-sensitive pathway is activated at the onset of brain injury as a protection to overcome oxidative stress. This was well demonstrated in tissue-culture studies showing that UCB-induced ROS excess impairs glutathione homeostasis [35, 40] and mitochondrial metabolism resulting in a cellular energetic crisis, swelling, cytochrome c release [41] and calcium perturbation [42]. All these events activate the mitochondrial apoptotic pathway [43] leading to increased oxidative stress and cellular redox status imbalance [44, 45]. Likewise, others and we showed that oxidative stress is a key feature of neurotoxicity animal models of systemic hyperbilirubinemia [18, 26, 46]. However, oxidative stress response to bilirubin did not elicit the induction of Cyps in our mouse model (Additional file 7B), differently to what observed in the Gunn rat [47], an animal model of the disease presenting a milder phenotype.

Interestingly, in our mouse model, the increase in ROS production resulted from the presence of activated microglia cells in the region where Purkinje cells degenerate. These cells, which are positive for HO-1, are involved in the removal of death cells and are associated with inflammation. We observed that *HO-1* expression levels increment over time in the damaged tissue, with HO1-positive microglia cells found as early as P5. This observation is in agreement Silva and colleagues showing that an excess of activated microglia produces high levels of nitric oxide, which in turn mediates microglia-induced neuronal cell death [48].

### ER stress response

The endoplasmic reticulum (ER) is very sensitive to perturbations of energy levels, intracellular calcium and redox state. Important modifications of its balance result in a condition named ER stress, characterized by an excess of unfolded proteins, and the induction of genes involved in cell survival and protein degradation, aiming to restore the normal state. However, when ER stress is sustained for long periods of time, cells are unable to overcome the accumulation of unfolded proteins and activate the apoptotic pathway.

We report that mRNA levels of key ER stress markers, such as *ATF3* and *Fas,* were significantly induced in response to high bilirubin levels. However, no changes in transcriptional levels were observed in other genes related to unfolded protein response (UPR) and ER stress (*GPR78, GRP94, P58* ^*IPK*^ *, FKBP11* and *DR5*) at any time point analysed (Additional file 6A). At the early stages of the disease, CHOP signal was increased in cerebellar sections of mutant mice, with more intense signal in the Purkinje cell layer. At later stages, CHOP signal was localized in degenerating Purkinje cells, surrounded by microglia Iba1-positive cells, suggesting their involvement in removing cellular debris from death Purkinje cells. These results are in agreement with previous findings performed in tissue culture cell lines [20, 35, 36], showing that CHOP and ATF3 are key mediators in ER-stress bilirubin-induced response.

Interestingly, the increased expression of ATF3, *Fas/CD95* and *CHOP* genes indicates that cells sense the overwhelming ER stress and activate the PERK-mediated pathway [49, 50]. Since bilirubin levels continuously increase over time [17], cells are not able to revert ER stress and activate the apoptotic pathway, a hypothesis supported by the increased levels of cleaved caspase 3 and TUNEL-positive cells observed in later stages of the disease [17].

Altogether, these observations provide evidence that ER stress is involved in the onset of bilirubin-induced neuro-apoptosis of Purkinje cells in vivo.

### PC neurodegeneration and neuro-apoptosis

The neurodegenerative phenotype in the cerebellum of the mice used in this study is progressively enhanced after sustained high levels of bilirubin and correlates well with the reduced cerebellar development (Fig. 1e). Neurodegeneration is associated with reduced arborization of PC and progressive cell loss (Fig. 1d). In contrast, mature granule cells were not affected by bilirubin toxicity (Fig. 1f). These results are in line with previous observations performed by our lab in the *Ugt1* ^*-/-*^ mouse model, in both C57Bl/6 and FVB/NJ genetic backgrounds [16–18]. In particular, we have previously demonstrated that PCs showed a neurodegenerative phenotype as determined by FluoroJade positivity, TUNEL assay and Sholl analysis [17, 18]. These data strengthen the concept that immature neurons are more sensitive to insults compared to more differentiated ones [51] and that bilirubin affects survival of neuronal precursors by impairing their neurogenesis, arborization and synaptic connectivity [17, 52].

### Inflammation: pro-inflammatory mediators

In the nervous system, degenerating cells can stimulate inflammation. Although most neurodegenerative diseases involve inflammation, also ER stress is a critical mechanism that can regulate inflammation and is linked through several intersecting cellular pathways, particularly those mediated by ROS [50].

We showed that *NFKβ*, a well-known inflammatory mediator, responds to high bilirubin levels by the upregulation of its mRNA in a whole tissue extract and by its increase in microglia cells which migrate to the site of tissue injury, together with the increase of TNFα in microglia and astrocytes. To this respect, NFKβ plays a dual role because it exerts both neurotoxic and neuroprotective functions depending on the kinetics and the expression in the injured tissue. In fact, NFKβ regulates the defence response to stress stimuli (inflammation and oxidative stress) and cellular fate (apoptosis, proliferation and differentiation). However, prolonged inflammation leads to the activation of genes, mediated by NFKβ that boosts oxidative stress, inflammation and apoptosis [50]. This is in line with our results showing the exacerbation of neurotoxicity during the more severe conditions observed at P10 (increase in *HO1*, caspase 3, astrocytosis and gliosis). Similarly, NFKβ is activated in response to pathological condition like trauma, ischemia or different types of neurodegenerative disease [39].

Another key inflammatory factor is TNFα, activated in the more severe phases of the pathology in the *Ugt1* ^*-/-*^ pups. This factor is released by activated microglia and astrocytes in the CNS and mediates the amplification of neuroinflammation events and the initiation of the apoptotic cascade. As in the cases of *NFKβ*, *TNFα* expression is increased in many neurodegenerative conditions [53].

Our findings support previous data by the laboratory of Brites and colleagues, showing that exposure of astrocytes and microglia primary cultures to bilirubin triggers the release of the pro-inflammatory cytokines TNFα and NFKβ [48, 54, 55]. However, in our model, increased transcriptional levels of *TNFα* and NFKβ occur after elevated and prolonged levels of tissue bilirubin, while in in vitro cultures these events occur within hours upon bilirubin induction. In addition, other well-known pro-inflammatory mediators are unchanged, such as *IL1β*, *IL6* and *INFγ* [56]. The discrepancy between in vitro and in vivo data might be related to intensity of the insult, interplay between different cell types and developmental status of the brain, conditions not fully recapitulated by in vitro primary cultures.

### Inflammation: cellular mediators

Activated microglia and astrocytes are the main effectors of the innate immune response in the CNS and are strongly activated in response to neurodegeneration, releasing neuroinflammatory factors. We showed that hyperbilirubinemia triggers the activation of both astrocytes and microglia in the cerebellum of *Ugt1* ^*-/-*^ mice.

The activation of both astrocytes and microglia by bilirubin has been reported both in vitro [57] and in vivo. In particular, in vivo studies of Gunn rat cerebellum indicated that the increase in GFAP content is a common trait of hyperbilirubinemia [58]. Moreover, the involvement of glia and the role of TLR2 were shown in the humanized knockout mouse model of hyperbilirubinemia [26, 59], although the time course of the disease onset was not investigated. The block of inflammatory activation resulted in death of hyperbilirubinemic *TLR2 KO* mice, underlined the beneficial effects of inflammatory response in the context of bilirubin toxicity [26]. However, despite the important inflammatory response observed in our *Ugt1* ^*-/-*^ animals, we could not detect *TLR2* activation in response to bilirubin (Additional file 5D). In fact, because of the early neonatal lethality of our mouse model, we investigated the developmental stages within P5 and P10, while *TLR2* activation was reported at later stages, namely P14 [26]. We speculate that the difference between the two models could be related to the extent and duration of the bilirubin insult, intrinsic differences between the two models, or a combination of both.

In addition, we investigated the interplay between inflammatory M1 and anti-inflammatory M2 microglia in the context of bilirubin toxicity. While M2 microglia was induced in at the early stages of the disease, decreasing over time as the severity of the phenotype increases, the trend of M1 microglia activation was the opposite, being progressively activated at later stages, as determined by Iba1 and *CD68* M1 microglia markers. A similar trend of activation/deactivation of M1 and M2 microglia was also observed in other neurodegenerative pathologies such as spinal cord injury [60] and traumatic brain injury [61, 62].

### Autophagy: the deathblow

Increasing evidences showed that autophagy is a crucial defence mechanism against neurodegenerative diseases and its deregulation is a key event that decides cell fate [63, 64].

Our results showed that autophagy is activated in response to bilirubin in the cerebellum of the *Ugt1* ^*-/-*^ mice at P10 (Fig. 8). These observations are in line with experiments performed in neuroblastoma and endothelial cell lines exposed to high bilirubin levels [35, 65]. In both cases, the autophagy pathway was activated at very high levels of bilirubin (Bf = 100–140 nM) and at later stages (24–72 h) when cell death was evident.

Interestingly, autophagy is linked to ER stress through Fas/CD95, which is strongly induced in the cerebellum of *Ugt1* ^*-/-*^ mice upon bilirubin exposure (Fig. 6). In fact, Fas/CD95 triggers cell death by the induction of caspase-mediated apoptosis but also stimulates autophagy through MAPK pathway in HeLa cells [66]. Moreover, CHOP is required to increase transcriptional levels of genes responsible for the correct formation and function of the autophagosome [67].

Taken together, these results indicate that autophagy is the latest pro-survival mechanism activated by cells to overcome bilirubin cellular stress prior to death.

## Conclusions

The dissection of the molecular mechanisms operating at the onset of neonatal hyperbilirubinemia revealed that different pathways are simultaneously regulated in response to the bilirubin insult, rather than being activated in a sequential time frame. Inflammation, together with ER stress, appear to be the leading processes resulting in neurodegeneration. The more precise understanding of the molecular interactions is crucial to set up therapeutic strategies.

To this respect 4-phenylbutyrate (4-PBA), an ER stress inhibitor, has been shown to reduce ER stress and neurodegeneration in a mouse model of Parkinson’s disease and tissue culture cells treated with bilirubin [36, 68]. Moreover, minocycline, a tetracycline with anti-inflammatory properties has been used in many neurodegenerative diseases to reduce inflammation and ameliorate the outcome of the disease [69], and in the Gunn rat, it reduced cerebellar abnormalities [24]. Administration of minocycline to juvenile Gunn rats resulted in the prevention of acute brainstem auditory evoked potential abnormalities produced by sulfadimethoxine [70, 71]. The concept that several components participate in the response to bilirubin neurotoxicity is supported by the observation that the treatment with antioxidants of Gunn rats having sulfadimethoxine-induced encephalopathy was not sufficient to reduce BIND. On the contrary, minocycline, a molecule with multiple properties in addition to the antioxidant effect, was able to prevent BIND [46]. It remains to be tested if the use of these compounds will ameliorate neurodegeneration and increase survival of the lethal mouse model presented in this study.

Further studies are needed to demonstrated whether modulation of one or more of them are required to reduce neurodegeneration and death, before this knowledge could be translated to patients.

## Additional files

Additional file 1: Table listing the oligonucleotides used. (XLSX 13 kb)

Additional file 2: **A)** Left panel, representative Nissl staining of cerebellar layers at P8 of WT and *Ugt1* ^*-/-*^ mice. Right panel, layer depth quantification (μm) at P8 and P10 of WT and *Ugt1* ^*-/-*^ mice. Scale bar 100 μm. **B)** WB analysis of total cerebellum protein extracts using an anti-NeuN antibody at P8. β-tubulin was used as a loading control. Values represent mean ± SD. **C)** Representative fluorescent immunohistochemistry of cerebellar sections from WT and *Ugt1* ^*-/-*^ mice using anti-NeuN antibody (green) to stain differentiated granule cells at P8. Hoechst (blue) was used to mark nuclei. Scale bar: 50 μm. For all the experiments the values represent the mean ± SD. Student *t* test, ns not significant; *p < 0.05, **p < 0.01. WT n = 4, *Ugt1* ^*-/-*^ n = 4. EGL, external germinal layer; IGL internal granular layer; ML, molecular layer. (TIF 6110 kb)

Additional file 3: **A)** Representative fluorescent immunohistochemistry of WT and *Ugt1* ^*-/-*^ cerebellum sections using anti-GFAP antibody (red) to highlight astrocytes, co-stained with an anti-calbindin antibody (green) to highlight PCs. Hoechst (blue) was used to mark nuclei. Scale bar: 500 μm. Boxed areas indicate fields shown in Fig. 3b. IV, VI, VIb, VII and IX indicate the cerebellar fissures. **B)** mRNA expression levels of *GFAP* at P5, P8 and P10 in total RNA preparations of WT and *Ugt1* ^*-/-*^ cerebella. For each gene, data were normalized according to the values of the WT samples at P5. Values represent the mean ± S.D. Two-way ANOVA, **p < 0.01, ***p < 0.001. Number of WT and *Ugt1* ^*-/-*^ was ≥3 in all the experiments. (TIF 11619 kb)

Additional file 4: **A)** mRNA expression levels of *Iba1* at P5, P8 and P10 in total RNA preparations of WT and *Ugt1* ^*-/-*^ cerebella. For each gene, data were normalized according to the values of the WT samples at P5. Values represent the mean ± S.D. One-way ANOVA test, *p < 0.05. **B)** Representative fluorescent immunohistochemistry of WT and *Ugt1* ^*-/-*^ cerebellum using an anti-Iba1 antibody (green) to highlight microglia, co-stained with an anti-GFAP antibody (red) to highlight astrocytes. Hoechst (blue) was used to mark nuclei. Scale bar: 500 μm. Boxed areas indicate fields shown in Fig. 3d. Number of WT and *Ugt1* ^*-/-*^ was ≥3 in all the experiments. (TIF 10480 kb)

Additional file 5: **A)** Example of expression levels under the detection limit of the technique. PCR product of *IL1β* mRNA expression. As positive control dendritic cells (DC) were treated with LPS for 24 hs. (lane 2). **B)** Example of detectable mRNA: *TNFα* expression. **C)** List of mRNAs that were not detected by qRT-PCR in cerebellar total RNA extracts. **D)** *TLR2* and **E)** *iNOS* mRNA relative expression levels in WT and *Ugt1* ^*-/-*^ animals at the indicated time points. For each gene, data were normalized according to the values of the WT samples at P5. Values represent mean ± SD. Two-way ANOVA, ns not significant. For representative agarose gels, lane 1: 1Kb ladder; lane 2: PCR product from dendritic cells (DCs) treated with LPS for 24 h; lane 3: CB, *Ugt1* ^*-/-*^ cerebellar total RNA extract; lane 4, blank. (TIF 2216 kb)

Additional file 6: **A)** Relative mRNA expression of ER stress response genes showing no changes at the different time points. For each gene, data were normalized according to the values of the WT samples at P5. Values represent the mean ± S.D. Two-way ANOVA, ns not significant. Number of WT and *Ugt1* ^*-/-*^ was ≥3 in all the experiments **B)** WB analysis and quantification of total cerebellum protein extracts of WT and *Ugt1* ^*-/-*^ mice using an anti-CHOP antibody at the indicated time points. Actin was used as loading control. Student *t* test, ns not significant. **C)** Representative IF of cerebellar sections from *Ugt1* ^*-/-*^ mice at P10 using an anti-CHOP antibody (red), co-stained with an anti-calbindin antibody (green) to highlight Purkinje cells; **D)** representative IF of cerebellar sections from WT and *Ugt1* ^*-/-*^ mice at P10 using an anti-CHOP antibody (red), co-stained with an anti-NeuN antibody to highlight granule cells; **E)** representative IF of cerebellar sections from *Ugt1* ^*-/-*^ mice at P10 using an anti-CHOP antibody (red), co-stained with an anti-Iba1 antibody to highlight microglia. For IF, Hoechst dye (blue) was used to mark nuclei. Scale bar: 50 μm. (TIF 24031 kb)

Additional file 7: Relative mRNA expression of **A)** oxidative stress response genes and **B)** *Cyp1a1* and *Cyp2a5* showing no changes at the different time points. For each gene, data were normalized according to the values of the WT samples at P5. Values represent the mean ± S.D. Two-way ANOVA, ns not significant. Number of WT and *Ugt1* ^*-/-*^ was ≥3 in all the experiments. **C)** Representative IF of cerebellar sections from WT and *Ugt1* ^*-/-*^ mice at P5 using an anti-HO1 antibody (red), co-stained with anti-Iba1 antibody to highlight microglia. For IF, Hoechst dye (blue) was used to mark nuclei. Scale bar: 50 μm. (TIF 10064 kb)

## Abbreviations

4-PBA: 4-phenylbutyrate
Arg1: Arginase 1
ATF3: Activating transcription factor 3
Bf: Free fraction of unbound bilirubin
BIND: Bilirubin-induced neurological dysfunction
CD68: Cluster of differentiation 68
CD86: Cluster of differentiation 86
CD95: Cluster of differentiation 95
Cyp1a2: Cytochrome P450 family 1 subfamily A member 2
Cyp2a5: Cytochrome P450 family 2 subfamily A member 5
EGL: External germinal layer
ER: Endoplasmic reticulum
GCs: Granule cells
GFAP: Glial fibrillary acidic protein
HO1: Heme oxygenase 1
Iba1: Ionized calcium-binding adapter molecule 1
IF: Immunofluorescence
IGL: Internal granular layer
IL18: Interleukin 18
IL1β: Interleukin 1β
LC3: Microtubule-associated protein 1A/1B-light chain 3
ML: Molecular layer
MMP2: Matrix-metallo-protease 2
MMP9: Matrix-metallo-protease 9
MRC1: Mannose receptor, C type 1
MRC2: Mannose receptor, C type 2
NFKβ: Nuclear factor kappa-light-chain-enhancer of activated B cells
nNOS: Neuronal nitric oxide synthase
Nrf2: Nuclear factor (erythroid-derived 2)-like 2
P: Post-natal day
PCR: Polymerase chain reaction
PCs: Purkinje cells
PT: Phototherapy
TNFα: Tumor necrosis factor alpha
Ugt1a1: UDP glucuronosyltransferase family 1 member A1
WB: Western blot
