# Supplementary figures and images for: Inflammatory signature of cerebellar neurodegeneration during neonatal hyperbilirubinemia in Ugt1-/- mouse model
Source: J Neuroinflammation. 2017 Mar 24;14:64. doi: 10.1186/s12974-017-0838-1 (PMC5366125; doi:10.1186/s12974-017-0838-1)

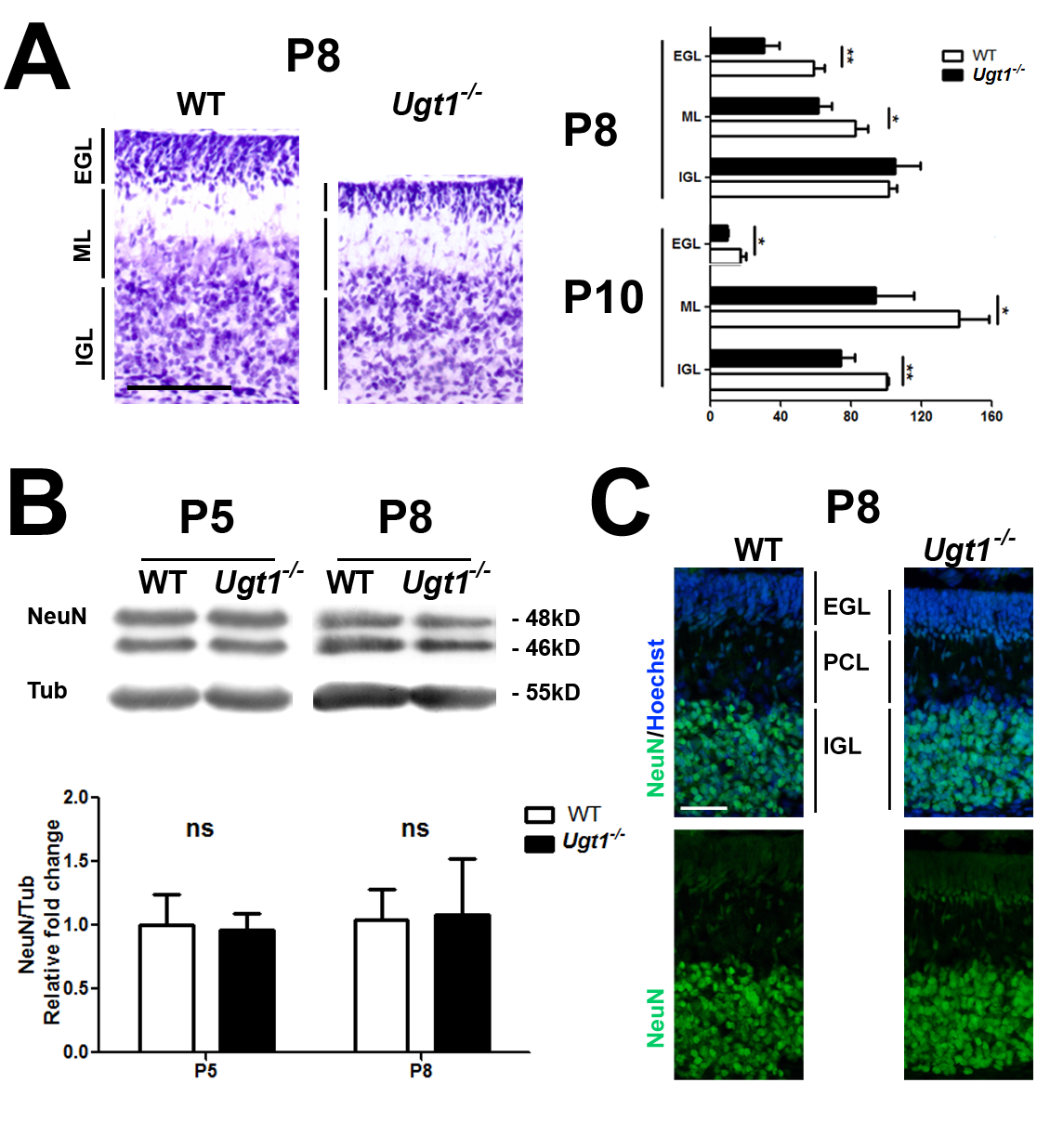

Supplement: Supplementary file 2 — A) Left panel, representative Nissl staining of cerebellar layers at P8 of WT and Ugt1 -/- mice. Right panel, layer depth quantification (μm) at P8 and P10 of WT and Ugt1 -/- mice. Scale bar 100 μm. B) WB analysis of total cerebellum protein extracts using an anti-NeuN antibody at P8. β-tubulin was used as a loading control. Values represent mean ± SD. C) Representative fluorescent immunohistochemistry of cerebellar sections from WT and Ugt1 -/- mice using anti-NeuN antibody (green) to stain differentiated granule cells at P8. Hoechst (blue) was used to mark nuclei. Scale bar: 50 μm. For all the experiments the values represent the mean ± SD. Student t test, ns not significant; *p < 0.05, **p < 0.01. WT n = 4, Ugt1 -/- n = 4. EGL, external germinal layer; IGL internal granular layer; ML, molecular layer. (TIF 6110 kb) [file 12974_2017_838_MOESM2_ESM.tif]

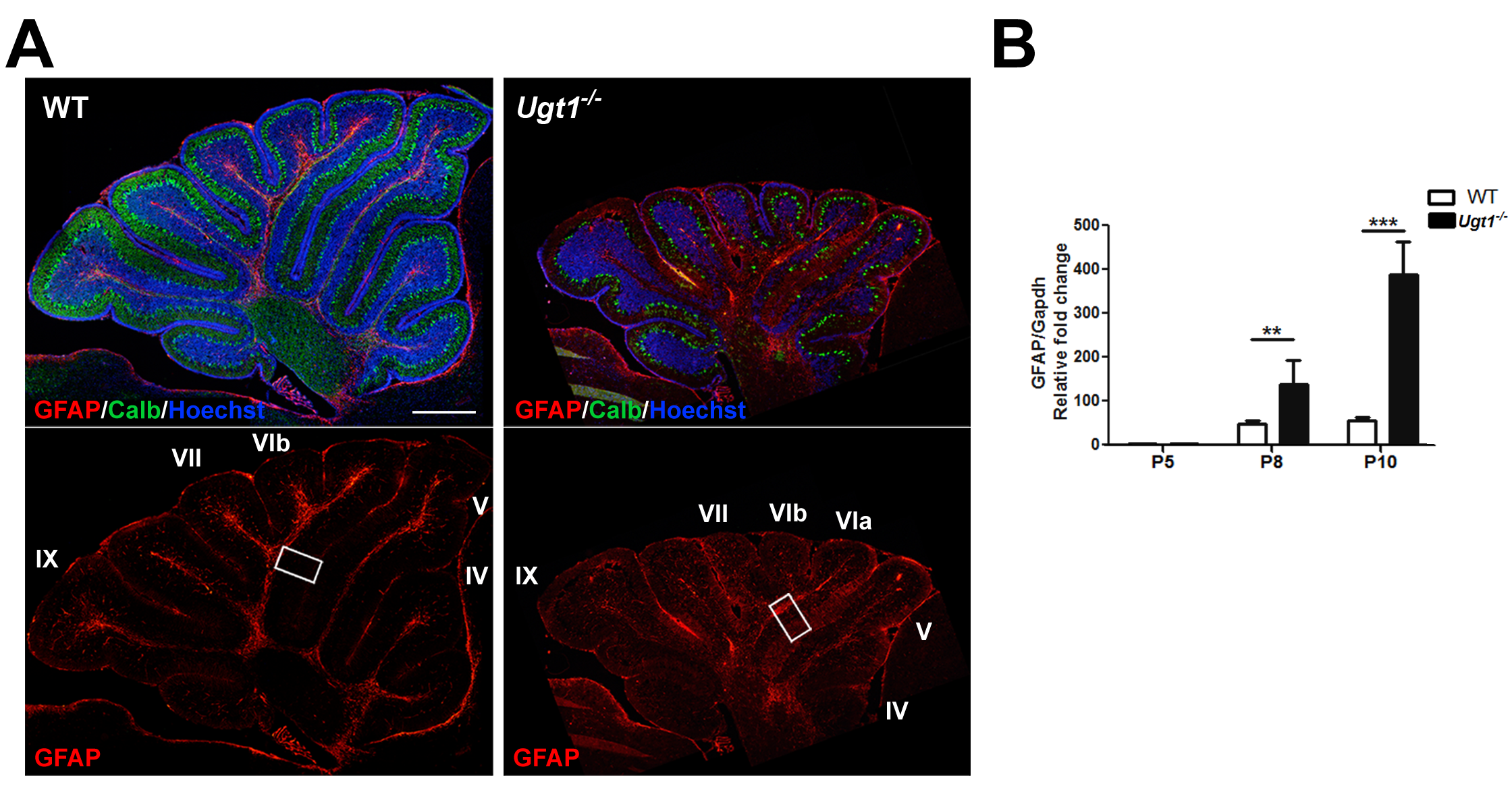

Supplement: Supplementary file 3 — A) Representative fluorescent immunohistochemistry of WT and Ugt1 -/- cerebellum sections using anti-GFAP antibody (red) to highlight astrocytes, co-stained with an anti-calbindin antibody (green) to highlight PCs. Hoechst (blue) was used to mark nuclei. Scale bar: 500 μm. Boxed areas indicate fields shown in Fig. 3b. IV, VI, VIb, VII and IX indicate the cerebellar fissures. B) mRNA expression levels of GFAP at P5, P8 and P10 in total RNA preparations of WT and Ugt1 -/- cerebella. For each gene, data were normalized according to the values of the WT samples at P5. Values represent the mean ± S.D. Two-way ANOVA, **p < 0.01, ***p < 0.001. Number of WT and Ugt1 -/- was ≥3 in all the experiments. (TIF 11619 kb) [file 12974_2017_838_MOESM3_ESM.tif]

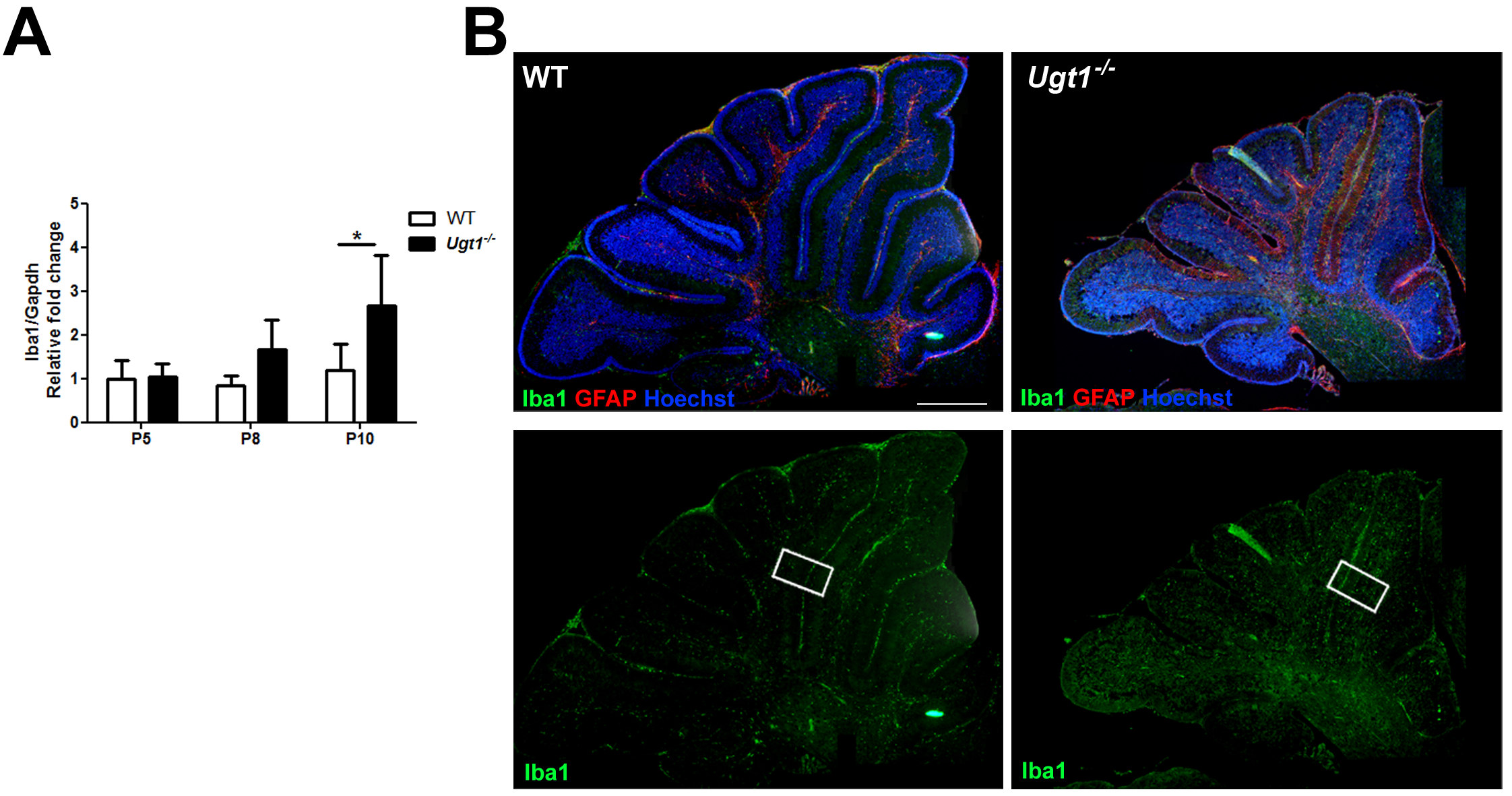

Supplement: Supplementary file 4 — A) mRNA expression levels of Iba1 at P5, P8 and P10 in total RNA preparations of WT and Ugt1 -/- cerebella. For each gene, data were normalized according to the values of the WT samples at P5. Values represent the mean ± S.D. One-way ANOVA test, *p < 0.05. B) Representative fluorescent immunohistochemistry of WT and Ugt1 -/- cerebellum using an anti-Iba1 antibody (green) to highlight microglia, co-stained with an anti-GFAP antibody (red) to highlight astrocytes. Hoechst (blue) was used to mark nuclei. Scale bar: 500 μm. Boxed areas indicate fields shown in Fig. 3d. Number of WT and Ugt1 -/- was ≥3 in all the experiments. (TIF 10480 kb) [file 12974_2017_838_MOESM4_ESM.tif]

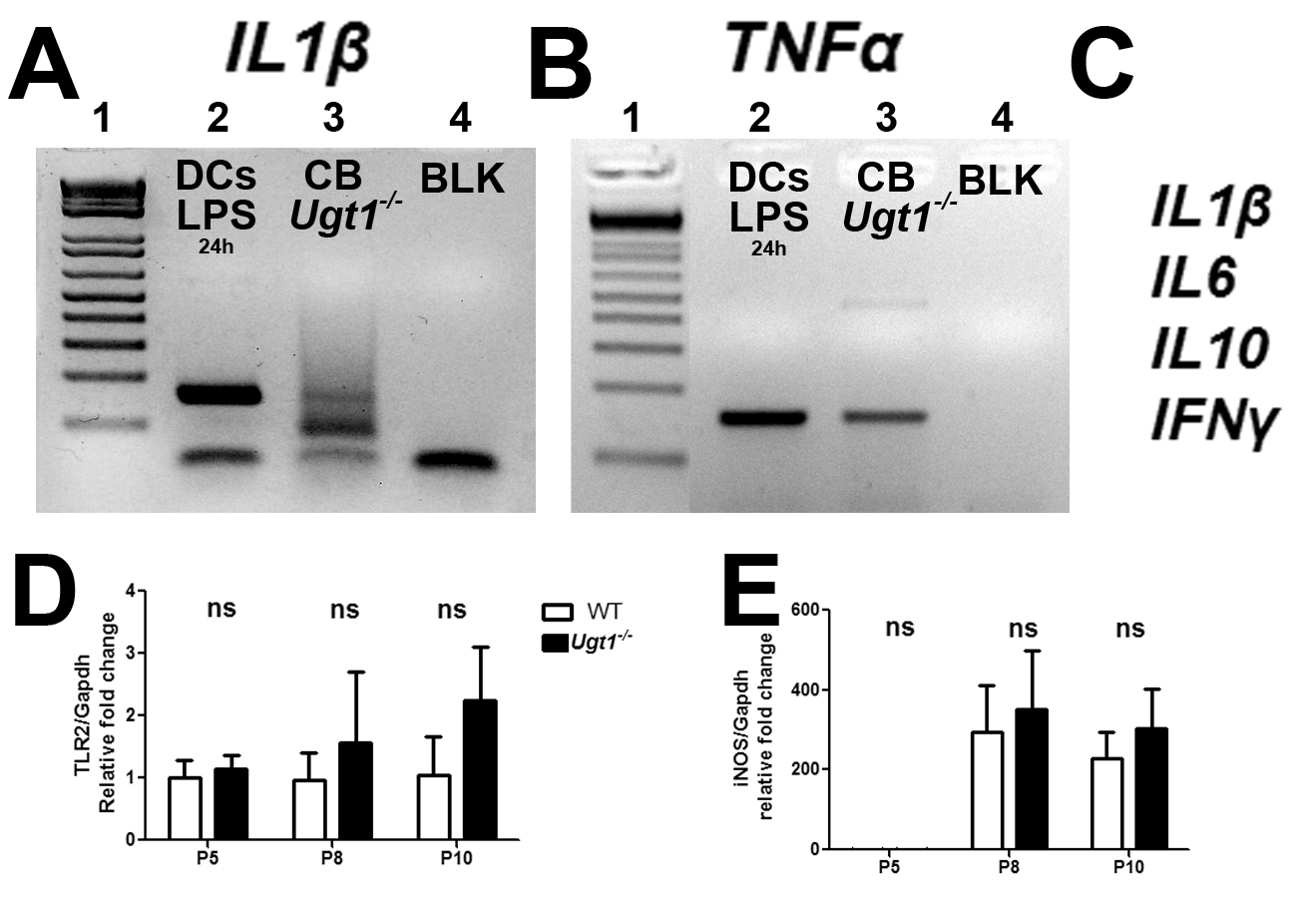

Supplement: Supplementary file 5 — A) Example of expression levels under the detection limit of the technique. PCR product of IL1β mRNA expression. As positive control dendritic cells (DC) were treated with LPS for 24 hs. (lane 2). B) Example of detectable mRNA: TNFα expression. C) List of mRNAs that were not detected by qRT-PCR in cerebellar total RNA extracts. D) TLR2 and E) iNOS mRNA relative expression levels in WT and Ugt1 -/- animals at the indicated time points. For each gene, data were normalized according to the values of the WT samples at P5. Values represent mean ± SD. Two-way ANOVA, ns not significant. For representative agarose gels, lane 1: 1Kb ladder; lane 2: PCR product from dendritic cells (DCs) treated with LPS for 24 h; lane 3: CB, Ugt1 -/- cerebellar total RNA extract; lane 4, blank. (TIF 2216 kb) [file 12974_2017_838_MOESM5_ESM.tif]

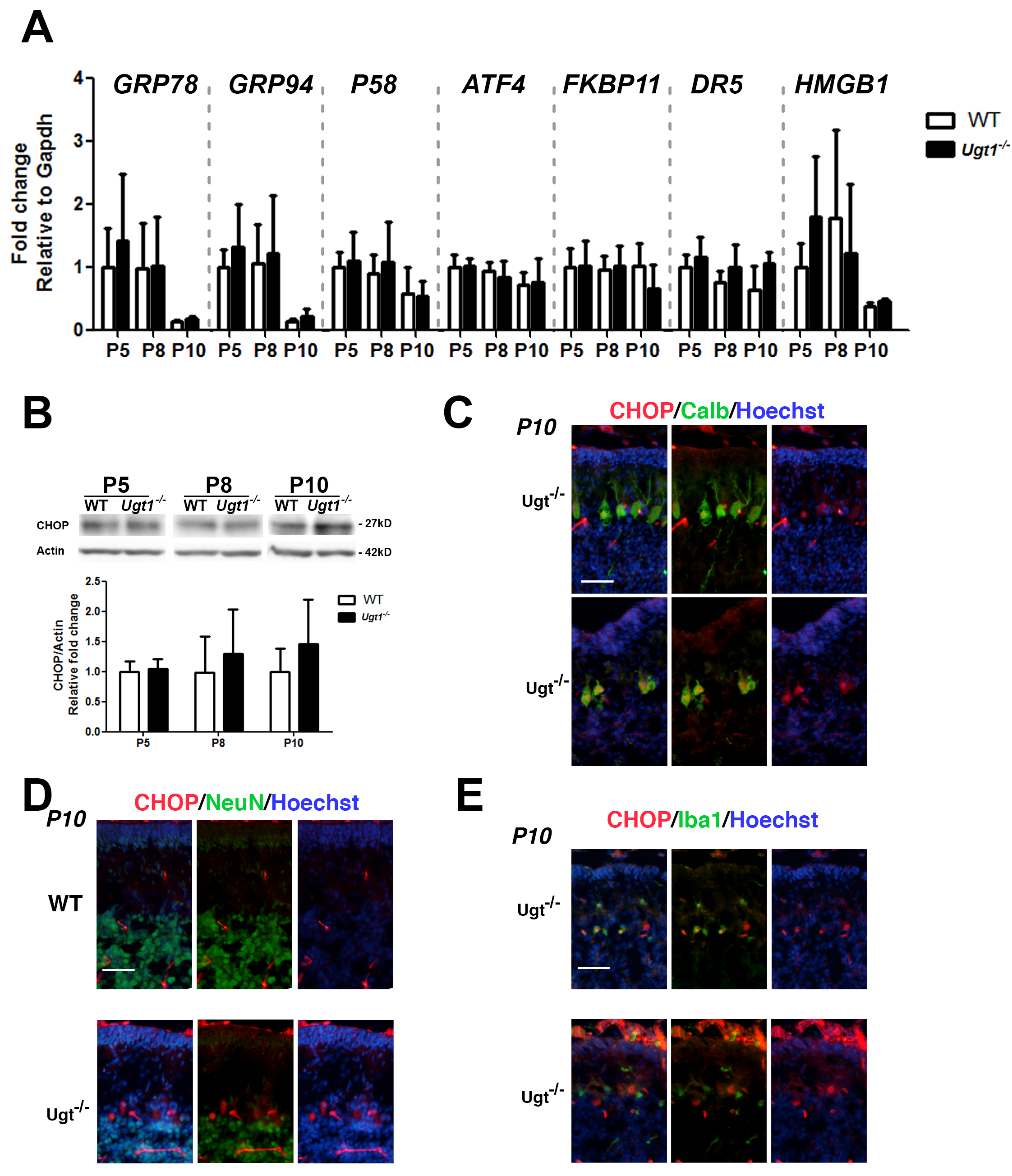

Supplement: Supplementary file 6 — A) Relative mRNA expression of ER stress response genes showing no changes at the different time points. For each gene, data were normalized according to the values of the WT samples at P5. Values represent the mean ± S.D. Two-way ANOVA, ns not significant. Number of WT and Ugt1 -/- was ≥3 in all the experiments B) WB analysis and quantification of total cerebellum protein extracts of WT and Ugt1 -/- mice using an anti-CHOP antibody at the indicated time points. Actin was used as loading control. Student t test, ns not significant. C) Representative IF of cerebellar sections from Ugt1 -/- mice at P10 using an anti-CHOP antibody (red), co-stained with an anti-calbindin antibody (green) to highlight Purkinje cells; D) representative IF of cerebellar sections from WT and Ugt1 -/- mice at P10 using an anti-CHOP antibody (red), co-stained with an anti-NeuN antibody to highlight granule cells; E) representative IF of cerebellar sections from Ugt1 -/- mice at P10 using an anti-CHOP antibody (red), co-stained with an anti-Iba1 antibody to highlight microglia. For IF, Hoechst dye (blue) was used to mark nuclei. Scale bar: 50 μm. (TIF 24031 kb) [file 12974_2017_838_MOESM6_ESM.tif]

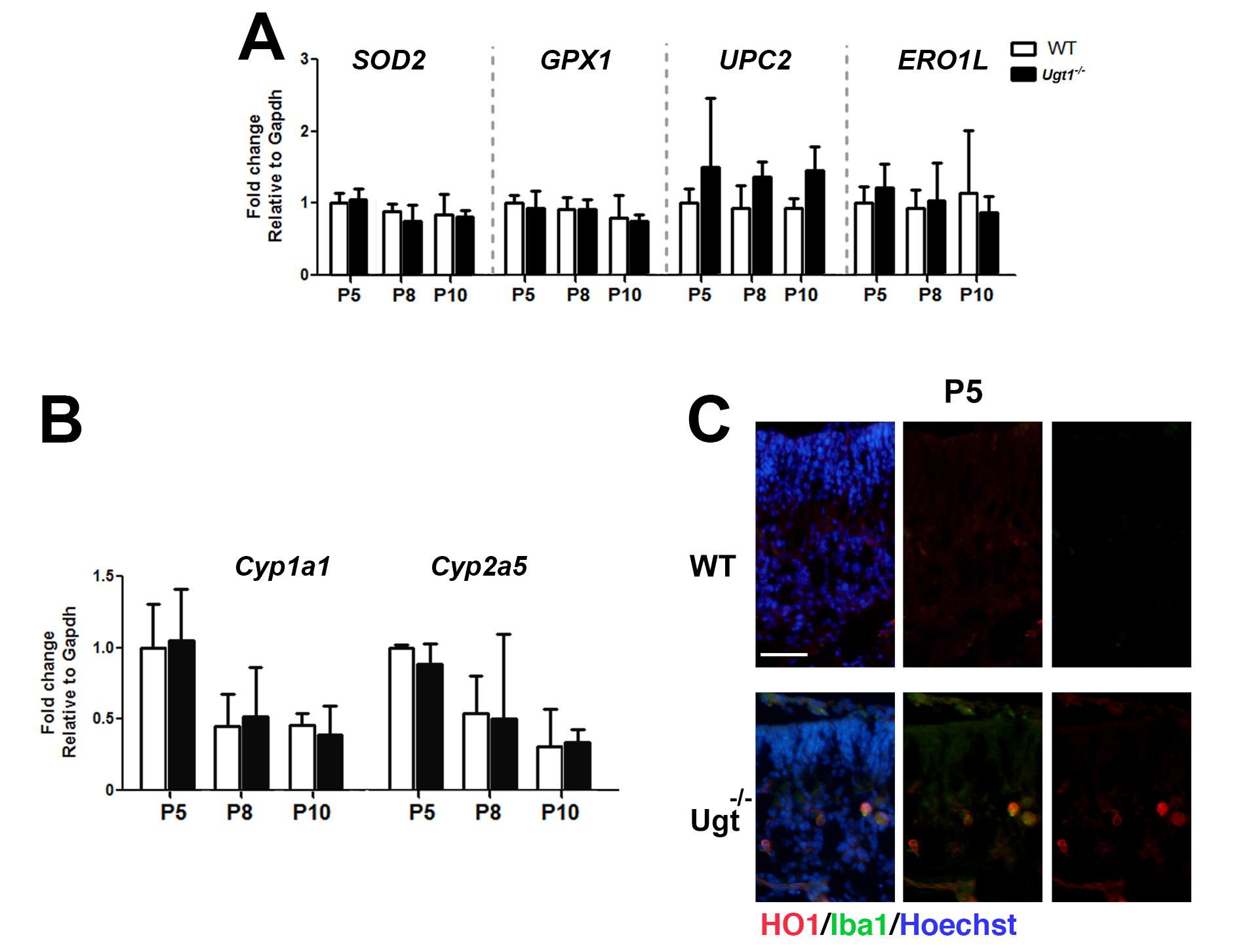

Supplement: Supplementary file 7 — Relative mRNA expression of A) oxidative stress response genes and B) Cyp1a1 and Cyp2a5 showing no changes at the different time points. For each gene, data were normalized according to the values of the WT samples at P5. Values represent the mean ± S.D. Two-way ANOVA, ns not significant. Number of WT and Ugt1 -/- was ≥3 in all the experiments. C) Representative IF of cerebellar sections from WT and Ugt1 -/- mice at P5 using an anti-HO1 antibody (red), co-stained with anti-Iba1 antibody to highlight microglia. For IF, Hoechst dye (blue) was used to mark nuclei. Scale bar: 50 μm. (TIF 10064 kb) [file 12974_2017_838_MOESM7_ESM.tif]
